# Evolution and Epidemiology of Multidrug-Resistant *Klebsiella pneumoniae* in the United Kingdom and Ireland

**DOI:** 10.1128/mBio.01976-16

**Published:** 2017-02-21

**Authors:** Danesh Moradigaravand, Veronique Martin, Sharon J. Peacock, Julian Parkhill

**Affiliations:** a Wellcome Trust Sanger Institute, Wellcome Genome Campus, Hinxton, Cambridgeshire, United Kingdom; b British Society for Antimicrobial Chemotherapy, Birmingham, United Kingdom; c Department of Medicine, University of Cambridge, Cambridge, United Kingdom; d London School of Hygiene and Tropical Medicine, London, United Kingdom; CDC

## Abstract

*Klebsiella pneumoniae* is a human commensal and opportunistic pathogen that has become a leading causative agent of hospital-based infections over the past few decades. The emergence and global expansion of hypervirulent and multidrug-resistant (MDR) clones of *K. pneumoniae* have been increasingly reported in community-acquired and nosocomial infections. Despite this, the population genomics and epidemiology of MDR *K. pneumoniae* at the national level are still poorly understood. To obtain insights into these, we analyzed a systematic large-scale collection of invasive MDR *K. pneumoniae* isolates from hospitals across the United Kingdom and Ireland. Using whole-genome phylogenetic analysis, we placed these in the context of previously sequenced *K. pneumoniae* populations from geographically diverse countries and identified their virulence and drug resistance determinants. Our results demonstrate that United Kingdom and Ireland MDR isolates are a highly diverse population drawn from across the global phylogenetic tree of *K. pneumoniae* and represent multiple recent international introductions that are mainly from Europe but in some cases from more distant countries. In addition, we identified novel genetic determinants underlying resistance to beta-lactams, gentamicin, ciprofloxacin, and tetracyclines, indicating that both increased virulence and resistance have emerged independently multiple times throughout the population. Our data show that MDR *K. pneumoniae* isolates in the United Kingdom and Ireland have multiple distinct origins and appear to be part of a globally circulating *K. pneumoniae* population.

## IMPORTANCE

*Klebsiella pneumoniae* is a major human pathogen that has been implicated in infections in healthcare settings over the past few decades. Antimicrobial treatment of *K. pneumoniae* infections has become increasingly difficult as a consequence of the emergence and spread of strains that are resistant to multiple antimicrobials. To better understand the spread of resistant *K. pneumoniae*, we studied the genomes of a large-scale population of extensively antimicrobial-resistant *K. pneumoniae* in the United Kingdom and Ireland by utilizing the fine resolution that whole-genome sequencing of pathogen genomes provides. Our results indicate that the *K. pneumoniae* population is highly diverse and that, in some cases, resistant strains appear to have spread across the country over a few years. In addition, we found evidence that some strains have acquired antimicrobial resistance genes independently, presumably in response to antimicrobial treatment.

## INTRODUCTION

*Klebsiella pneumoniae* is a common environmental human- and animal-associated Gram-negative bacterium that has become a major cause of nosocomial infections worldwide (1–4). Commonly found as a commensal bacterium, *K. pneumoniae* has the potential to cause a wide range of infections, including soft tissue, wound, and respiratory tract infections, in particular in patients with a compromised immune system (3). In addition to numerous reports of nosocomial infections, *K. pneumoniae* has been observed to cause community-acquired infections (1, 3). *K. pneumoniae* infections are frequently detected as outbreaks in health care settings, in particular in neonatal units (5). *K. pneumoniae* can be transferred through medical equipment and blood products (6, 7) and can be carried within the intestinal tracts of patients and on the skin surface of hospital personnel (3, 8, 9). *K. pneumoniae* can also cause invasive disease in various animal species (10, 11), and animal and food sources have been proposed to serve as potential reservoirs for *K. pneumoniae* (12).

In keeping with the high microbial diversity of the niches that *K. pneumoniae* occupies, recent studies have shown that this species has a flexible and diverse pangenome containing numerous accessory genes that enable the bacterium to adapt to various habitats and respond to environmental stresses such antibiotic treatment (4). Holt et al. (4) generated a global phylogeny of *K. pneumoniae* isolates from environmental and hospital sources and demonstrated that in the light of detailed phylogenetic evidence, the *K. pneumoniae* species complex may be split into three distinct species referred to as *K. pneumoniae* (KpI), *K. quasipneumoniae* (KpII), and *K. variicola* (KpIII), all of which are known to cause infections in humans (13, 14) and each of which contains a high level of diversity. Since then, the number of clinical genomic studies of *K. pneumoniae* has increased. While some of these studies have reported the rapid spread of particular strains of *K. pneumoniae* across a region/country (15–17), others have focused on outbreaks in single hospitals (18, 19). In either case, the investigations commonly identify multidrug-resistant (MDR) strains, in particular, carbapenem-resistant strains.

The treatment of *K. pneumoniae* infections has become more difficult as a result of the emergence of these MDR lineages of *K. pneumoniae*. These lineages carry a wide range of antimicrobial resistance genes that restrict the available options to effectively treat *K. pneumoniae* infections. Known mechanisms of resistance include the production of β-lactamases such as extended-spectrum β-lactamases (ESBLs), cephalosporinases, and carbapenemases (20–24). The spread of resistance is linked to mobile genetic elements that may also carry virulence determinants that enhance the ability of the bacterium to colonize and establish infection within the host (3, 25). These factors include the capsule, various adhesins required for adherence of the bacterium to host tissues, and siderophores for iron absorption (26, 27).

Because of the growing importance of MDR *K. pneumoniae*, it is important to understand its population structure and the relationship between this and the genetic diversity of antibiotic resistance. However, while we now have a better understanding of the global diversity of this species and outbreaks in single hospitals, the origin and national transmission of MDR *K. pneumoniae* involved in hospital infections remain largely unknown. To address this, we used genomics and phylogenetic analysis to investigate a systematic collection of MDR *K. pneumoniae* isolates obtained from hospitals across the United Kingdom and Ireland over the past decade. In particular, we analyzed the structure of this population in the context of a recently published major collection of *K. pneumoniae* to elucidate the position and recent emergence of United Kingdom and Ireland MDR isolates from within the global population. In addition, we systematically identified the distribution of known genes encoding virulence factors and antibiotic resistance determinants within the population.

## RESULTS

**FIG 1:**
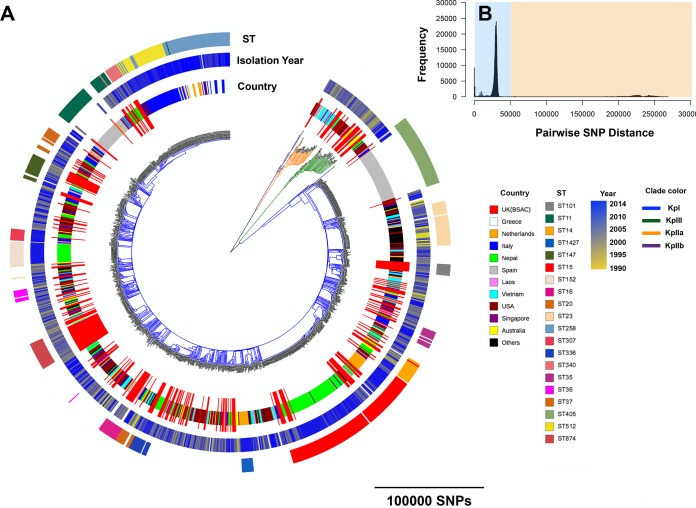
(A) A neighbor-joining tree based on 1,000,726 SNPs and constructed for the combined United Kingdom and global collections with their STs and years of isolation. Only STs that were represented by at least 10 isolates in the population are shown. The United Kingdom (BSAC) isolates are the United Kingdom and Ireland MDR isolates. (B) The distribution of pairwise SNP differences in the United Kingdom samples and the global collection. The blue and yellow backgrounds correspond to the different levels of divergence discussed in the text.

We first placed the MDR *K. pneumoniae* isolates from the United Kingdom and Ireland collection in the context of the global population structure. The resulting combined phylogenetic tree revealed that the United Kingdom and Ireland MDR isolates reside on multiple branches of the global tree but some clades are composed mainly of United Kingdom samples (Fig. 1A). It is also evident that the United Kingdom and Ireland MDR *K. pneumoniae* population is as diverse as the global population, indicating that lineages found within the United Kingdom and Ireland collection may have multiple sources outside the United Kingdom. As shown previously for the global collection of Holt et al. (4), the MDR United Kingdom collection contains deep divisions between the major species of *K. pneumoniae*. The KpI, KpIIA, KpnIIB, and KpIII species are all represented in the United Kingdom and Ireland collection, with the KpI species predominating (Fig. 1A). In line with Holt et al. (4), we also identified distinct peaks, representing two distinct time scales of phylogenetic divergence, in the pairwise single-nucleotide polymorphism (SNP) distance distribution (Fig. 1B). The peaks of >50,000 SNPs with the yellow background in Fig. 1B correspond to the divergence between the KpI, KpIIA, KpIIB, and KpIII species. In contrast, the other peaks, shown with a blue background in Fig. 1B, correspond to distinct groups of long and short branches primarily within the KpI species and in some cases within the KpII and KpIII species.

The distribution of pairwise SNP distances for isolates of the same or different sequence types (STs) indicated that the resolution of multilocus sequence typing (MLST) is largely limited to SNP distances of >10,000 SNPs (see Fig. S1A in the supplemental material). Mapping of the STs of United Kingdom and Ireland MDR *K. pneumoniae* isolates onto the phylogenetic tree revealed a high level of concordance between MLST and the whole-genome sequence-based phylogeny, demonstrating the discriminatory power of MLST in detecting major clades (Fig. S1B). MLST analysis of the United Kingdom and Ireland isolates demonstrated a variety of STs, with ST15 being the most common (Fig. S1C). Some of the STs present in our collection, such as ST15 and ST147, are known to be linked with multidrug resistance worldwide (28, 29) and in particular in Europe (30).

10.1128/mBio.01976-16.1 FIG S1  (A) The distribution of pairwise SNP difference for the United Kingdom and Ireland MDR and global collections for the blue region shown in Fig. 1B. Identical and different STs are red and blue, respectively. The nonidentical SNP frequencies are normalized by the number of identical SNPs to better present the accuracy of MLST. (B) Distribution of STs across the phylogenetic tree for the United Kingdom collection. (C) The MLST compositions of the United Kingdom and global collections. Singleton STs are not shown. Only STs represented by at least 10 isolates in the population are shown. Download FIG S1, PDF file, 0.2 MB. Copyright © 2017 Moradigaravand et al. 2017 Moradigaravand et al. This content is distributed under the terms of the Creative Commons Attribution 4.0 International license.

**FIG 2:**
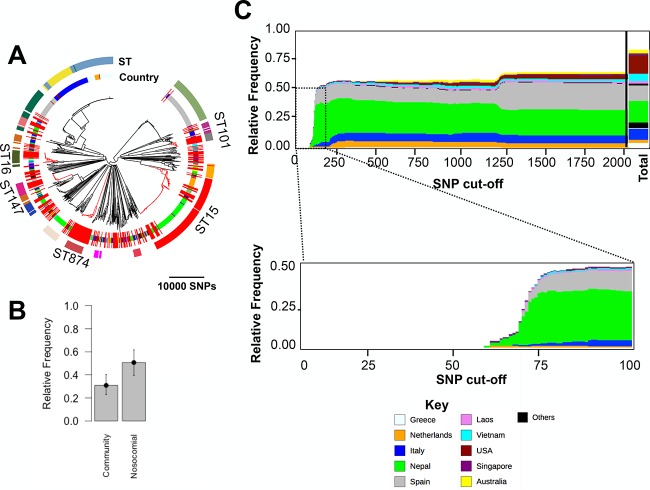
(A) A neighbor-joining phylogenetic tree for the KpI lineage depicting the linkages between isolates in the United Kingdom and Ireland collection and those in the global collection with ≤10,000 SNPs divergence from them (equivalent to membership in the same ST). The outer circular band shows STs that were represented by at least 10 isolates, and the next band inward shows the country of origin according to the key in Fig. 1. The clades in red are the major STs that contained at least 10 United Kingdom and Ireland MDR isolates. (B) The relative frequencies of global isolates in the phylogenetic tree in panel A based on the source of acquisition of infection for those isolates in the global study of Holt et al. (4). The error bars denote 95% confidence intervals obtained from the binary distribution. (C) The origin of the closest non-United-Kingdom relatives of United Kingdom and Ireland isolates in the global population shown at increasing SNP cutoffs from left to right. The total column represents the proportion of isolates in the combined tree. United Kingdom and Ireland MDR *K. pneumoniae* isolates are not shown.

To identify potential introductions to, and subsequent spread of lineages within, the United Kingdom and Ireland MDR isolate collection, we next focused on divergences between isolates of the same ST, i.e., isolates that were <10,000 SNPs apart, within the MDR United Kingdom and Ireland isolates and between the MDR United Kingdom and Ireland isolates and the global isolates (Fig. 2A). (Note that we did not remove recombined regions at this stage, so this value includes SNPs due to point mutations, as well as those introduced by recombination.) To this end, we redrew the tree by including all of the United Kingdom and Ireland isolates and any isolates from both collections that were <10,000 SNPs distant from a United Kingdom and Ireland isolate. The resulting tree showed that a majority of the isolates belonging to the United Kingdom and Ireland collection clustered in closely related clades, with isolates from the global collection often basal to, but sometimes interspersed within, these clades (Fig. 2A). Using the detailed information about the infectious source of *K. pneumoniae* for the Holt et al. (4) isolates, we found that isolates of nosocomial origin seem to be overrepresented (i.e., appear more frequently to be related to the United Kingdom and Ireland isolates than would be expected from their frequencies in the Holt et al. (4) collection [Fig. 2B]) in comparison with community-acquired *K. pneumoniae*. Moreover, we found some isolates of nonhuman (monkey, bovine, and mouse) origins and nonhuman clinical isolates spread across this tree. This suggests that animals and the environment may serve as hidden reservoirs of clinical MDR *K. pneumoniae*, as has been proposed for food animals and retail meat (12).

In order to further analyze the relationship between the United Kingdom and Ireland MDR isolates and the global collection, we constructed multiple trees consisting of the United Kingdom and Ireland isolates and any isolate from the global collection that was within a specified SNP distance of a United Kingdom isolate for a range of SNP distances. We then calculated the proportion and origin of global isolates that appeared in the trees at each SNP distance cutoff. The results demonstrated that trees limited to the most closely related isolates consisted entirely of United Kingdom and Ireland isolates, while global isolates from Nepal, the Netherlands, Italy, and Spain appeared sequentially as the cutoffs were widened (Fig. 2D). Although this may be driven to some extent by the biases in the collection, we note that the most frequently sampled sites are not necessarily those that appear closest to the United Kingdom and Ireland isolates. These connections demonstrate frequent direct or indirect transmissions between the United Kingdom and Ireland and mainly European but also geographically very distant countries, indicating that United Kingdom and Ireland MDR *K. pneumoniae* isolates are part of globally circulating lineages.

**FIG 3:**
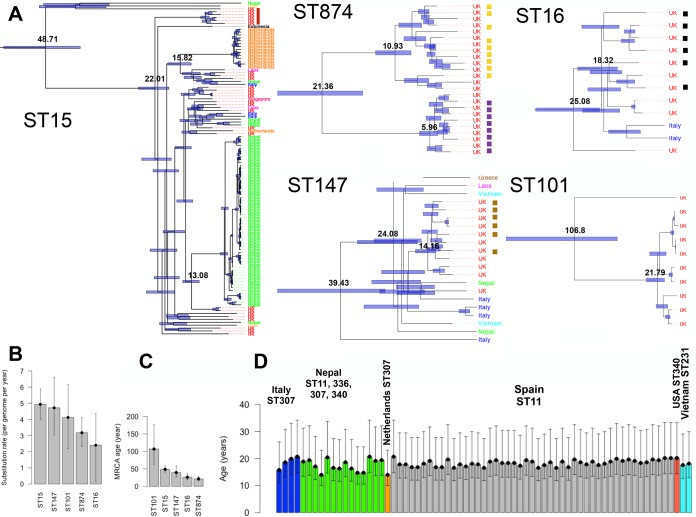
(A) Dated Bayesian phylogenies for the United Kingdom and Ireland STs that contain at least 10 United Kingdom and Ireland MDR *K. pneumoniae* isolates. The bars on the nodes show 95% confidence intervals. The values on the nodes denote divergence times in years. The colored bars in the columns next to each clade show putative outbreaks in the same hospitals. The most recent collection years of isolates in the ST15, ST147, ST874, ST101, and ST16 clades were 2013, 2012, 2009, 2010, and 2013, respectively. (B) Substitution rates estimated for the clades in panel A. The error bars denote the 95% confidence interval. (C) The age of the MRCA of the clades in panel A. The error bars denote 95% confidence intervals. (D) Dates of the MRCA of United Kingdom and Ireland isolates and global isolates for the isolates not present in the major ST clades shown in panel A. The estimated age of divergence between United Kingdom and global isolates for linkages within a cutoff of 80 SNPs corresponds to ~20 years. This age and the upper and lower values of the error bar (95% confidence interval) were obtained by dividing the pairwise SNP distances by the mean substitution rate and also the upper and lower 95% confidence interval bounds for the substitution values in panel B.

The phylogenetic tree in Fig. 2A consists of few major STs that were more frequent in the population than other STs. We identified the clades that correspond to ST15, ST16, ST147, ST101, and ST874, which contained at least 10 United Kingdom and Ireland MDR *K. pneumoniae* isolates and exhibited various degrees of geographic heterogeneity. Except for the ST874 clade, which contained one ST103 isolate, the other clades were homogeneous and comprised only a single ST. We used Bayesian dated phylogenetic analysis in BEAST to compute the substitution rate of the genomes and estimate the dates of the most recent common ancestor (MRCA) of the United Kingdom and Ireland MDR isolates and their closest non-United-Kingdom isolates in each clade (Fig. 3A to C). The substitution rate showed some variation across the clades, but the average was ~3.8 SNPs per genome per year (6.34 × 10^−7^ SNPs per site per year) (Fig. 3B). The dated phylogenetic trees revealed that a majority of the clades were formed within the past few decades (Fig. 3A and C). The ST101 and ST874 clades consisted entirely of United Kingdom isolates but formed >100 years ago and 20 years ago, respectively. While ST101 isolates originated from various hospitals in the United Kingdom and Ireland, the ST874 clade included two linked putative outbreaks in two different hospitals (Fig. 3A). The ST16 clade with an MRCA of 25 years predominantly comprises an outbreak in the United Kingdom and isolates from sporadic infections of bla_OXA-48_ carbapenemase-producing isolates in two hospitals in Spain (15) and two isolates from Italy (because of the unavailability of exact isolation years, the Spanish isolates were not included in the Bayesian tree of ST16 in Fig. 3A). The ST147 and ST15 clades were more geographically diverse and had MRCAs of 39 and 48 years, respectively. The ST147 clade included four MDR isolates from two hospitals in Italy (16) and some isolates from Vietnam, Laos, and Nepal that are linked with a putative outbreak caused by the United Kingdom and Ireland MDR isolates. The ST15 clade is highly diverse, consisting of linked outbreaks caused by the United Kingdom and Ireland MDR isolate collection and previously reported ones in Nepal and the Netherlands (18, 19, 31), which diverged from the United Kingdom and Ireland MDR isolates 13 and 15 years ago, respectively (Fig. 3A). Altogether our results demonstrate that the United Kingdom and Ireland MDR *K. pneumoniae* isolates in these major STs are connected to global isolates, which are sometimes from outbreaks of carbapenemase-encoding and MDR strains. Since our collection is extensively susceptible to imipenem, the connection to these outbreaks suggests a potential risk of the rise of carbapenem-resistant strains within hospitals in the United Kingdom in the near future. To generalize the findings from the major STs, we first excluded isolates of major STs from the population and then used the average substitution rate to obtain the origins of the apparent transmissions from the global collection to the United Kingdom and Ireland that occurred within the last ~20 years, i.e., since the formation of the ST874 clade as the most recent clade. The results showed that apparent recent divergences on this time scale involved isolates from several countries (Fig. 3D). In particular, several isolates of ST307 from Italy and Nepal showed recent divergences from a single MDR United Kingdom and Ireland isolate. Furthermore, two MDR United Kingdom and Ireland isolates appeared to be from the ST11 clade, which is composed of isolates from the recent spread of a carbapenem-resistant lineage across Spain, further underlining the risk of the spread of carbapenem-resistant strains to United Kingdom hospitals.

The genomic diversity of the MDR United Kingdom *K. pneumoniae* collection was also evident in the size of the accessory genome. The number of genes in the core genome, i.e., the number of genes shared by >99% of the isolates, was 2,958, which is higher than the 1,743 reported for the global collection (4), probably a reflection of the higher diversity in that collection. The soft-core genome (genes shared by >95% and <99% of the isolates) had an additional 543 genes. The noncore genome (genes shared by >0% and <95% of the isolates) was very large, consisting of 25,044 genes. This large dispensable genome was also partly due to the presence of multiple plasmids in the isolates. The isolates predominantly harbored plasmids with Kpn3, FIIK, PKP91, and Col replicons (Fig. S2). Of these, FIIk, FIBk, and Kpn3 have been reported to carry multiple beta-lactamases, particularly cephalosporinases, and are often shared by members of the family *Enterobacteriaceae* (32, 33).

10.1128/mBio.01976-16.2 FIG S2  The distribution of plasmid replicons across the phylogenetic tree for the United Kingdom and Ireland MDR strain collection. Plasmid replicons that are present in fewer than five isolates are not shown. Download FIG S2, PDF file, 0.3 MB. Copyright © 2017 Moradigaravand et al. 2017 Moradigaravand et al. This content is distributed under the terms of the Creative Commons Attribution 4.0 International license.

Infection by *K. pneumoniae* involves a number of host interaction or virulence factors, including capsule production proteins, fimbriae, lipopolysaccharides, siderophores, and efflux pumps (26). Our data show that the capsule gene (*wzi*), the aerobactin siderophore receptor gene (*iutA*), and the fimbrial gene (*mrk*) were frequently present in every isolate (Fig. S3). In contrast, the yersiniabactin gene (*ybt*), iron transporter permease genes (*kfuB* and *kfuC*), and iron regulatory protein genes (*irp1* and *irp2*), all involved in iron metabolism, were present in only a proportion of the isolates contained in certain clades (Fig. S3A), showing that isolates in these clades are developing various iron uptake mechanisms and may have the potential to turn into novel hypervirulent *K. pneumoniae* strains (Fig. S3A). The higher diversity of siderophores has been suggested to make bacteria more capable of taking up environmental iron by evading larger numbers of non-siderophore-producing cheater bacteria (34). The higher efficiency of iron uptake may result in phenotypic changes such as higher capsule production, involved in the formation of hypervirulent phenotypes (27, 35, 36). Furthermore, the number of virulence genes varies across major STs and appeared to be relatively higher in ST101, ST336, and ST29, which provides genomic evidence of differential virulence characteristics (Fig. S3B).

10.1128/mBio.01976-16.3 FIG S3  (A) The distribution of virulence factor genes across the phylogenetic tree for the United Kingdom and Ireland MDR isolate collection. Genes that are present in fewer than four isolates are not shown. (B) The number of virulence genes within STs represented by at least eight isolates in the data set. The values above the boxes are the numbers of isolates. The boxes show the 0.75 and 0.25 quartiles of the data subsets. Download FIG S3, PDF file, 0.1 MB. Copyright © 2017 Moradigaravand et al. 2017 Moradigaravand et al. This content is distributed under the terms of the Creative Commons Attribution 4.0 International license.

The availability of quantitative MICs, rather than qualitative resistant/susceptible classifications, allowed us to directly identify genetic determinants that cause increased antibiotic resistance in MDR *K. pneumoniae* (Fig. S4). As expected, we found a high correlation between MICs of antibiotics with similar mechanisms of action, such as minocycline and its derivative tigecycline and cefuroxime/cefotaxime (Fig. S5A). The phenotypic results indicated that the population was broadly resistant, i.e., >50% of the population, to the penicillins amoxicillin (susceptible [S], 0; intermediate [I], 0; resistant [R], 250), amoxicillin-clavulanate (S, 78; I, 0; R, 172), and piperacillin-tazobactam (S, 58; I, 72; R, 120); the cephalosporins ceftazidime (S, 78; I, 11; R, 161), cefotaxime (S, 77; I, 1; R, 147), and cefuroxime (S, 15; I, 0; R, 235); ciprofloxacin (S, 62; I, 24; R, 164); and the aminoglycoside gentamicin (S, 116; I, 0; R, 134). In contrast, the collection was less resistant to imipenem, the carbapenem antibiotic used in this study (S, 248; I, 2; R, 0), as well as tigecycline (S, 120; I, 72; R, 51) (Fig. S5B). We found that increased resistance to most antibiotics has emerged throughout the tree, suggesting that the ongoing evolution of MDR *K. pneumoniae* clades is driven by antibiotic treatment (Fig. S5C). Among the major STs identified here, ST101 and ST147 were more resistant to tetracycline and ST101 and ST340 were more resistant to cefotaxime and ciprofloxacin, as shown by the distribution of MICs across the major STs (Fig. S5D).

10.1128/mBio.01976-16.4 FIG S4  A comparison of the EUCAST distribution and the BSAC collection for the antibiotics studied here. Abbreviations: amoxicillin, amx; cefuroxime, cxm; amoxicillin-clavulanate, amc; cefotaxime, ctx; cefoxitin, fox; imipenem, ipm; piperacillin-tazobactam, tzp; ciprofloxacin, cip; ceftazidime, caz; gentamicin, gen; tigecycline, tgc; minocycline, min; tetracycline, tet. The dashed vertical lines show the clinical breakpoints for antibiotics with known values. The curves illustrate the density function fitted to the distributions. Download FIG S4, PDF file, 0.3 MB. Copyright © 2017 Moradigaravand et al. 2017 Moradigaravand et al. This content is distributed under the terms of the Creative Commons Attribution 4.0 International license.

10.1128/mBio.01976-16.5 FIG S5  (A) The antibiogram for antibiotics with known clinical breakpoints. (B) The correlations between the MICs of various classes of antibiotics shown as a correlogram. Blue and red represent positive and negative correlations, respectively. The abbreviations are the same as those in Fig. 4. (C) Distribution of MICs and resistance values across the phylogenetic tree. The abbreviations are the same as in Fig. S4. (D) A comparison of MICs across various STs. The abbreviations are the same as in Fig. S4. (E) MICs of the beta-lactam antimicrobials for the ESBL and non-ESBL subpopulations. The boxes show the 0.75 and 0.25 quartiles of the data subsets and the whiskers represent 1.5 times the InterQuartile Range, or the maximum value, where this does not exceed 1.5x the IQR. The abbreviations are the same as those in Fig. S4. Download FIG S5, PDF file, 0.9 MB. Copyright © 2017 Moradigaravand et al. 2017 Moradigaravand et al. This content is distributed under the terms of the Creative Commons Attribution 4.0 International license.

*K. pneumoniae* resistance to beta-lactam drugs is attributed primarily to the presence of ESBLs (3). The MICs of amoxicillin-clavulanate, ceftazidime, and cefuroxime for the ESBL-producing isolates in our collection were higher than those for the non-ESBL-producing isolates (Fig. S5E), and these isolates appeared to produce various beta-lactamases, some of which are known to lead to the ESBL-producing phenotype. Most notably, the *bla*_CTX-M-15_ gene, which has been reported to be prevalent across Europe (37), and *bla*_SHV_ variants SHV-12, SHV-39, SHV-100, and SHV-40 were gained by isolates across the tree (Fig. S6A). These genes, along with rare putative ESBL-encoding genes such as *bla*_CTX-M-26_, *bla*_SHV-27_, and *bla*_SHV-5_, account for 95% of the ESBL phenotype, and some of these were strongly associated with elevated levels of resistance to beta-lactams (Fig. S6B). These variant beta-lactamase-encoding genes were acquired multiple times throughout the tree as accessory genes and were associated with increased resistance to amoxicillin, ceftazidime, and cefuroxime in isolates with elevated MICs (Fig. S6C). Besides ESBLs, resistance to cephalosporins and cephamycins may be mediated by AmpC beta-lactamases in members of the family *Enterobacteriaceae*. Although *K. pneumoniae* is known to lack the chromosomally located *ampC* gene, eight variants of *ampC* in the accessory genome seem to have been sporadically acquired by isolates across the phylogenetic tree. An extra copy of the *ampC* gene is exclusively present in one isolate, i.e., 12045_7#42, with a read coverage of 40× across the whole gene, which has the highest MIC of cefoxitin, as the only cephamycin studied here. The gene appears to be linked with phage proteins and occurred in the context of some *K. pneumoniae* plasmids. The variance in the MICs of cefoxitin for other isolates might be attributable to other resistance mechanisms, such as differential beta-lactamase expression levels. Despite several reports of outbreaks of carbapenemase-producing *K. pneumoniae*, carbapenem resistance is relatively uncommon in Western Europe (38), and this holds true for our collection. In a single isolate that exhibited a high MIC of imipenem (8 μg/ml), the *bla*_NDM1_ gene, which encodes a dominant *Enterobacteriaceae* carbapenemase first detected in India and Pakistan, was present (39).

10.1128/mBio.01976-16.6 FIG S6  (A) Distribution of major beta-lactamase-encoding genes across the phylogenetic tree. (B) Frequency of beta-lactamase-encoding genes in the pangenome. The annotated functions of the genes can be found in the list of genes in the pangenome deposited in a public repository (for the link, see Materials and Methods). (C) Distribution of putative resistance genes, the presence of which strongly correlate with the MICs across the phylogenetic tree for the beta-lactams. The *bla_2*, *group_8659*, and *group_1309* genes are copies of *bla*_TEM1_, *bla*_CTX-M-15_, and *bla*_SHV_ genes, respectively. The sequences of these genes have been deposited in a public repository (for the link, see Materials and Methods). (D) Frequency of potential major tetracycline resistance genes, i.e., genes present in more than five isolates, in the pangenome. The annotated functions of the genes can be found in the list of genes in the pangenome deposited in a public repository (for the link, see Materials and Methods). (E) Distribution of putative resistance the presence of which strongly correlates with MICs across the phylogenetic tree. The *group_9260*, *tetD_2*, *yedA_2*, *tetR*, and *tetA_2* genes encode the tetracycline repressor protein TetR, tetracycline resistance protein efflux class D, a drug/metabolite transporter permease, a TetR family transcriptional regulator, and tetracycline efflux protein TetA, respectively. The sequences of these genes have been deposited in a public repository (for the link, see Materials and Methods). Download FIG S6, PDF file, 0.3 MB. Copyright © 2017 Moradigaravand et al. 2017 Moradigaravand et al. This content is distributed under the terms of the Creative Commons Attribution 4.0 International license.

The expression of tetracycline efflux pumps is a known resistance mechanism across a wide range of species (40). In our isolates, in addition to the chromosomally located *tetA* and *tetD* tetracycline efflux protein-encoding genes, which were found in the core genome, additional copies of *tetA* and *tetD* had been acquired by multiple isolates, and these appeared to be associated with increased MICs (Fig. S6D and E).

Three classes of aminoglycoside-modifying enzymes (adenylyltransferases, phosphotransferases, and acetyltransferases) generally confer gentamicin resistance on members of the family *Enterobacteriaceae*. A number of isolates spread throughout the tree appeared to have independently acquired different gentamicin resistance-encoding genes, which included mainly aminoglycoside acetyltransferase [*aac*(*6′*) in 125 isolates and *aac*(*3*)*-II* in 97 isolates]-, adenylyltransferase (*aadA* in 93 isolates)-, and phosphotransferase (*strA* and *strB* genes in 83 isolates and *aph*(*3′*)*-I* in 46 isolates)-encoding genes. Similar to gentamicin, ciprofloxacin is reported to still be an effective treatment for *K. pneumoniae* infections, although the rate of ciprofloxacin resistance in *K. pneumoniae* has been rising recently (41, 42). Ciprofloxacin resistance is generally mediated through mutations in the *gyrB* and *gyrA* (gyrase) genes and the *parC* (topoisomerase IV) gene. In our collection, the *gyrB* E468D nonsynonymous mutation, previously reported to increase the MIC by 8-fold, had arisen as the result of two different point mutations in four and six isolates and was strongly associated with elevated ciprofloxacin MICs (*P* < 0.0001 [Student’s *t* test]) (43).

## DISCUSSION

We used genomic and phylogenetic approaches to analyze a collection of MDR *K. pneumoniae* isolates systematically obtained from bloodstream infections in hospitals across the United Kingdom and Ireland. In particular, we studied the population structure and variation of this collection in the phylogenetic context of other global *K. pneumoniae* collections to uncover the specific relationships between United Kingdom and Ireland isolates and global isolates. In addition, the availability of drug susceptibility (MIC) data allowed us to identify genetic determinants associated with antibiotic resistance.

Our findings indicate that the United Kingdom and Ireland *K. pneumoniae* population is highly diverse, encompassing isolates from the major lineages of *K. pneumoniae* and various STs, some of which have been associated with the global dissemination of *K. pneumoniae* (28). In particular, we found that several clones of MDR *K. pneumoniae* have emerged recently and spread across the country and in some cases have given rise to outbreaks. Furthermore, there were apparent links between United Kingdom and Ireland isolates and outbreaks in mainly European hospitals. We noted that the closest relatives of United Kingdom and Ireland MDR *K. pneumoniae* were more likely to be isolates from nosocomial rather than community-acquired infections. This highlights the importance of identifying putative reservoirs of *K. pneumoniae* that may be involved in the transmission of *K. pneumoniae* between distant hospitals and thus lead to the global circulation of *K. pneumoniae*. As a commensal opportunistic pathogen, *K. pneumoniae* has the potential to spread rapidly between hospitals via carriage in patients transferred between countries, medical tourists, or blood products (44). Further tracing of patients involved in intercountry transmissions could help to find any missing intermediates in the transmission chain and, by doing so, determine the extent of direct versus indirect hospital transmission.

We found that genetic determinants that increased the resistance level (MIC) have emerged across the population. This finding, along with the observation that United Kingdom and Ireland MDR isolates were nested within the global collection, demonstrates that even already MDR lineages can become more resistant and can disseminate rapidly. The introduction of ESBL-producing *K. pneumoniae* into Europe, and on some occasions into the United Kingdom, has been ascribed to the transfer of patients from countries where MDR *K. pneumoniae* is endemic (38, 45). This is particularly concerning for the introduction of carbapenem resistance from outside the United Kingdom, as this is one of the few remaining antibiotic classes that are effective against *K. pneumoniae* infections. Our study provides further evidence of the independent emergence of resistant lineages due to the acquisition of determinants of resistance to currently effective antibiotics (39).

The high resolution of whole-genome sequencing enabled us to elucidate the fine structure of the United Kingdom and Ireland MDR *K. pneumoniae* population in this study. Using a geographically broader collection of clinical drug-sensitive, as well as resistant, *K. pneumoniae* would allow an understanding of the extent of the global network of interhospital transmissions and also identify previously unrecognized sources and reservoirs of this pathogen. This will be essential for designing effective means to bring the dissemination of this infection under control.

## MATERIALS AND METHODS

### Isolates and antibiotic susceptibility testing.

This study was approved by the National Research Ethics Service (reference no. 12/EE/0439) of the United Kingdom and the Cambridge University Hospitals Research and Development Department. Two hundred fifty *K. pneumoniae* isolates were collected by the British Society for Antimicrobial Chemotherapy (BSAC). The collection was composed of isolates that were submitted to a systematic bacteremia surveillance program between 2001 and 2011 by 28 hospitals across the United Kingdom and Ireland. The *K. pneumoniae* collection was derived from a large-scale systematic collection of Gram-negative MDR pathogens from a selection of hospitals across the United Kingdom and Ireland chosen to maximize geographic diversity. In order to maximize temporal diversity, isolates were taken from each of the 10 years of sampling. This yielded a temporally and geographically diverse collection of MDR isolates. We then analyzed all of the *K. pneumoniae* isolates in this collection, which were spread across the majority of the hospitals over 10 years. A list of isolates in the collection is provided in Table S1.

10.1128/mBio.01976-16.8 TABLE S1  Accession numbers, years, countries of isolation, and MICs of different antibiotics for isolates in the United Kingdom and Ireland MDR *K. pneumoniae* collection. Download TABLE S1, CSV file, 0.04 MB. Copyright © 2017 Moradigaravand et al. 2017 Moradigaravand et al. This content is distributed under the terms of the Creative Commons Attribution 4.0 International license.

We defined multidrug resistance as nonsusceptibility to three or more classes of antimicrobials, as described in reference 46. Isolates were collected if they were resistant to at least one antibiotic in three of the following classes: penicillins, carbapenems, cephalosporins, tetracyclines, aminoglycosides, and fluoroquinolones.

To contextualize our isolates, we used sequence data from a previously published global collection that contains genomes of isolates from animals and humans with both environmental and nosocomial infection sources, mainly from five countries across the world (4). To maximize the diversity of the contextual population, we included a further nine published clinical collections of *K. pneumoniae* recovered mainly from Europe but also from Asia and America in our analysis (15, 16, 18, 19, 31, 47–50). The collection includes any previously published data sets with more than 10 assemblies submitted to the NCBI and two further data sets from Nepal. The accession and study numbers of each, as well as the country of origin, are shown in Table 1.

**TABLE 1:** *K. pneumoniae* isolates used in this study to contextualize the United Kingdom and Ireland MDR *K. pneumoniae* collection

Study no.	PubMed ID no.	Country
ERP000165	26100894	Multiple
PRJEB1272	26769896	Spain
PRJNA252925	26864946, 26617589	Netherlands
PRJEB1800	25712531	Nepal
PRJEB7967	26199326	Multiple
PRJEB6543	25367909	Italy
PRJNA267549	26230489	United States
PRJEB7661	26135860	Italy
PRJEB10561	26817488	Greece
PRJNA253300	25267672	Nepal

The agar dilution method was employed to obtain the MIC of each antibiotic for each isolate. The antibiotics included penicillins (amoxicillin, amoxicillin-clavulanic acid, and piperacillin-tazobactam), cephalosporins (cefuroxime, cefotaxime, and ceftazidime), a cephamycin (cefoxitin), an aminoglycoside (gentamicin), a fluoroquinolone (ciprofloxacin), tetracyclines (minocycline and tetracycline), and a glycylcycline (tigecycline) (46). The distribution of MICs for our samples was compared with the distributions from the European Committee on Antimicrobial Susceptibility Testing (EUCAST). The clinical resistance breakpoints were downloaded from the EUCAST website (http://www.eucast.org) on 15 March 2016.

### Sequencing and pangenome analysis.

DNA extraction was performed with the QIAxtractor (Qiagen) instrument according to the manufacturer’s instructions. Illumina sequencing libraries with a 450-bp insert size were prepared according to the manufacturer’s protocols and sequenced on an Illumina HiSeq2000 with paired-end reads with a length of 100 bp. Ninety-six samples were multiplexed per lane to give an average depth of coverage of ~90-fold. We assembled paired-end sequence reads by employing an assembly and improvement pipeline (51) that is based on Velvet (52) and subsequently annotated the *de novo* assemblies with Prokka (53). To perform the pangenome analysis, we took the output from Prokka and analyzed it with Roary (54).

### Phylogenetic analysis and substitution rate calculation.

We mapped the short reads against the reference genome of *K. pneumoniae* Ecl8 (GenBank accession no. HF536482 and CANH01000000) with SMALT v 0.7.4 (https://www.sanger.ac.uk/resources/software/smalt/). We employed a conservative minimum score of 30 for mapping and then annotated SNPs with a combination of SAMtools mpileup (55) and BCFtools. We removed the SNPs at sites with heterogeneous mapping in which the SNP was present in less than 75% of the reads at that site, similar to reference 56.

The multiple alignment was used to obtain the global tree. To estimate the substitution rate within each major clade, i.e., ST15, ST101, ST147, ST16, and ST874 (see Results for more details), we first selected the isolate with the best contig statistics (i.e., with the largest N50 value and lowest contig number) from that clade in our collection. We then joined the contigs and used the resulting pseudogenome as a local reference genome and mapped the reads from each clade to this. We obtained multiple alignments of SNP sites for each clade by the method described above. Subsequently, we eliminated high-density SNP regions that had undergone recent recombination by using Gubbins, which detects recombination by using SNP density (57). The recombinations occurred primarily in hot spots that included phage and transposon genes and occasionally genes coding for membrane and capsular proteins, such as the *wzi* gene. The ST15, ST101, ST147, ST16, and ST874 clades contained 2, 3, 5, 2, and 2 phage regions, respectively. The SNPs that occurred in these regions appeared to account for the majority (between 90 and 95%) of the SNPs that accumulated in the major clades. The ST15, ST101, ST147, ST16, and ST874 clades had 21,316, 9,436, 27,626, 4,480, and 2,315 variant sites before the removal of hypervariable sites, respectively. Gubbins reduced these to 2,302, 588, 1,150, 358, and 279 variants, respectively.

We then used the multiple alignments to obtain phylogenetic trees and used the trees to plot the root-to-tip distance versus the time of isolation for each clone. To assess the significance of the temporal signal, i.e., the clock-like accumulation of mutations over time, we conducted 10,000 bootstraps with randomized years to obtain a distribution for R-squared values. We then compared the real R-squared values with the distribution. We found a strong temporal signal at >99% confidence for the ST147 clade and >85% for the ST15 and ST874 clades. The temporal signals were weaker (>60%) for the ST16 and ST101 clades. To estimate the substitution rate, we used BEAST v 1.7 (58). We examined various models, including a strict molecular clock and a lognormal model with a constant population size. We used the results of the lognormal model, as it was favored by the maximum-likelihood test (with 500 bootstraps) conducted with the Tracer software of the BEAST package.

We ran three independent chains of BEAST for 50 million generations with sampling every 10 generations. Convergence was tested by using effective sample sizes that had to be >200 for key parameters to confirm convergence. Ten million states were excluded as the burn-in phase, and the output trees were then merged to attain a dated tree with the TreeAnnotator software from the BEAST package. We used in-house tools, FigTree (tree.bio.ed.ac.uk/software/figtree), and iTOL (59) to visualize the results of phylogenetic analysis.

### *In silico* MLST analysis and identification of antimicrobial resistance determinants, virulence factors, and plasmids.

We used the srst2 package (60), with a 90% coverage cutoff, to map the short reads to antibiotic resistance genes, virulence genes, and plasmid replicons. The resistance gene and plasmid replicon databases were obtained from the srst2 package. For virulence genes, we used the database of the Pasteur Institute (http://bigsdb.web.pasteur.fr/klebsiella/klebsiella.html). The results were visualized on the phylogenetic tree with an in-house tool. The STs were determined with an *in silico* MLST pipeline that takes assemblies and compares them against allele data derived from the public MLST database at http://www.pubmlst.org/kpneumoniae. We identified ESBLs as defined in the Lahey Hospital and Medical Centre database of beta-lactamases and ESBLs, which is available at http://www.lahey.org/Studies.

### Regression analysis and identification of antibiotic resistance determinants.

In addition to screening the database of known resistance genes, we developed a genome-wide statistical approach to identify genes/SNPs that are strongly associated with elevated MICs as quantitative values rather than categorical resistant/susceptible values. In doing so, we utilized the higher variance in MICs than in categorical resistance status to identify genetic determinants that underlie the increase in the MICs of specific antibiotics. This approach is particularly useful for understanding mechanisms of resistance to antibiotics like tetracycline, for which no clinical breakpoint is available.

To this end, we first developed a multiple regression model in the form of MIC ~ Gene_(0/1)_ + ST, where MIC is a continuous dependent variable that corresponds to the MIC of each antibiotic and Gene_(0/1)_ denotes the presence and absence of the individual accessory genes (the output of Roary). (Note that, to preform the linear regression model, we made the approximation assumption that MICs are continuous.) To account for the population structure, we also included the ST information, given the high level of concordance between ST clusters and major phylogenetic clades, as a categorical predictor variable. We then identified the genes that generated a significantly positive slope coefficient (95% confidence interval) for the Gene_(0/1)_ variable. Subsequently, we filtered the genes with *P* values of <10^−4^ (Student’s *t* test) for the t statistic of association for the Gene_(0/1)_ variable, which measures the significance that the slope coefficient was greater than 0. In addition, we set a *P* < 0.05 filter for the *F* test, which tests the overall significance of the whole regression test. Finally, we ranked the hits on the basis of the *P* values and the R-adjusted value; for a list of hits with their association values, see Table S2, and for the results, see Fig. S7A. These values are depicted in Fig. S10 for the different antibiotics we studied here.

10.1128/mBio.01976-16.7 FIG S7  Plots of adjusted R-squared values for the regression models versus the −log_10_ *P* value for the t statistics obtained from Student’s *t* test for the Gene(0/1) (A) and SNP(0/1) (B) variables for different antibiotics. The points in red circles correspond to resistance gene/SNPs discussed throughout this report. Only genes or SNPs with *P* values of <10^−4^ are shown. Download FIG S7, PDF file, 0.3 MB. Copyright © 2017 Moradigaravand et al. 2017 Moradigaravand et al. This content is distributed under the terms of the Creative Commons Attribution 4.0 International license.

10.1128/mBio.01976-16.9 TABLE S2  The hit list of the accessory genes identified by regression analysis of the accessory genes and their regression statistics for different antibiotics. Abbreviations: amoxicillin, amx; cefuroxime, cxm; amoxicillin-clavulanate, amc; cefotaxime, ctx; cefoxitin, fox; imipenem, ipm; piperacillin-tazobactam, tzp; ciprofloxacin, cip; ceftazidime, caz; gentamicin, gen; tigecycline, tgc; minocycline, min; tetracycline, tet. Download TABLE S2, CSV file, 0.2 MB. Copyright © 2017 Moradigaravand et al. 2017 Moradigaravand et al. This content is distributed under the terms of the Creative Commons Attribution 4.0 International license.

In the second regression model, we first identified annotated SNPs in the core genome after mapping the reads to the reference genome of *K. pneumoniae* Ecl8 and removing SNP sites where >5% of the reads had an N at that site. As above, we developed a regression model in the form of MIC ~ SNP_(0/1)_ + ST, where the MIC and ST variables are defined as mentioned above. The SNP_(0/1)_ variable is the predictor variable and represented the presence or absence of individual SNPs. Similar filter values were used to identify SNPs, the presence of which was strongly associated with MICs, and the hit list and statistical parameters are detailed in Table S3 and Fig. S7B. This model was particularly used to study ciprofloxacin resistance, which is often conferred by chromosomal mutations.

10.1128/mBio.01976-16.10 TABLE S3  The hit list of individual SNPs identified by SNP-based regression analysis and their regression association statistics for different antibiotics. The abbreviations of the antibiotic names are the same as those in Table S2. Download TABLE S3, CSV file, 3 MB. Copyright © 2017 Moradigaravand et al. 2017 Moradigaravand et al. This content is distributed under the terms of the Creative Commons Attribution 4.0 International license.

### Data availability.

To allow the retrieval of sequences of genes in the accessory genome, we have deposited these sequences, as well as the full list of genes in the pangenome, in a public repository (https://data.mendeley.com/datasets/xfw8n3wzs5/1).
