# Supplementary figures and images for: Evolution and Epidemiology of Multidrug-Resistant Klebsiella pneumoniae in the United Kingdom and Ireland
Source: mBio. 2017 Feb 21;8(1):e01976-16. doi: 10.1128/mBio.01976-16 (PMC5358916; doi:10.1128/mBio.01976-16)

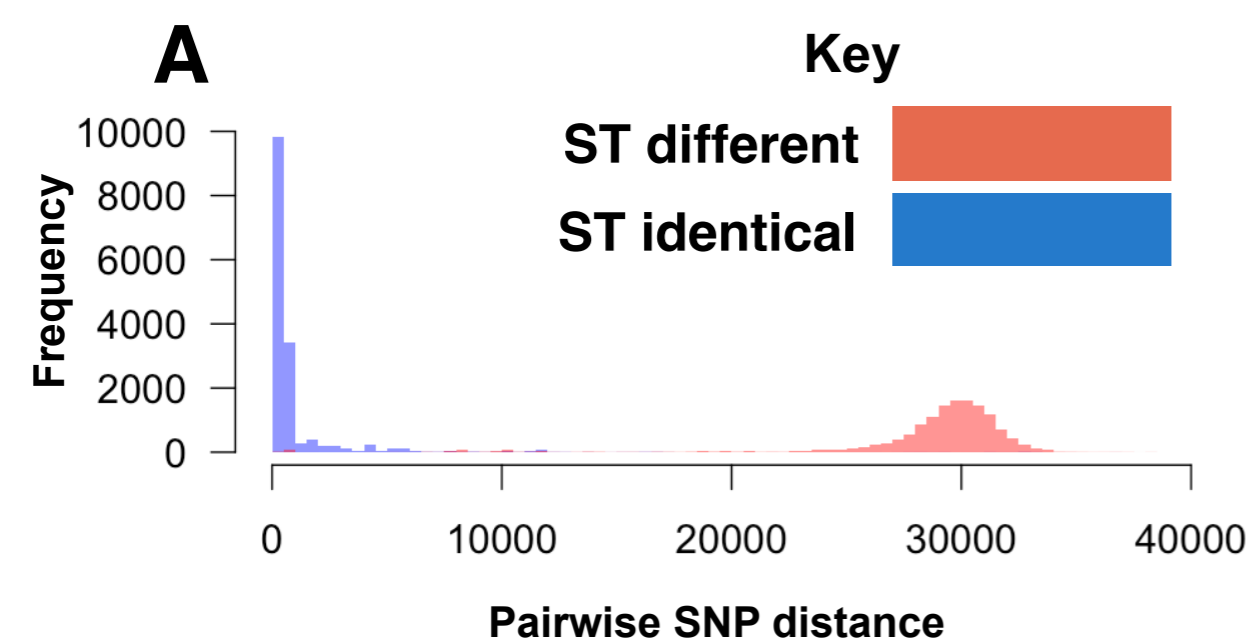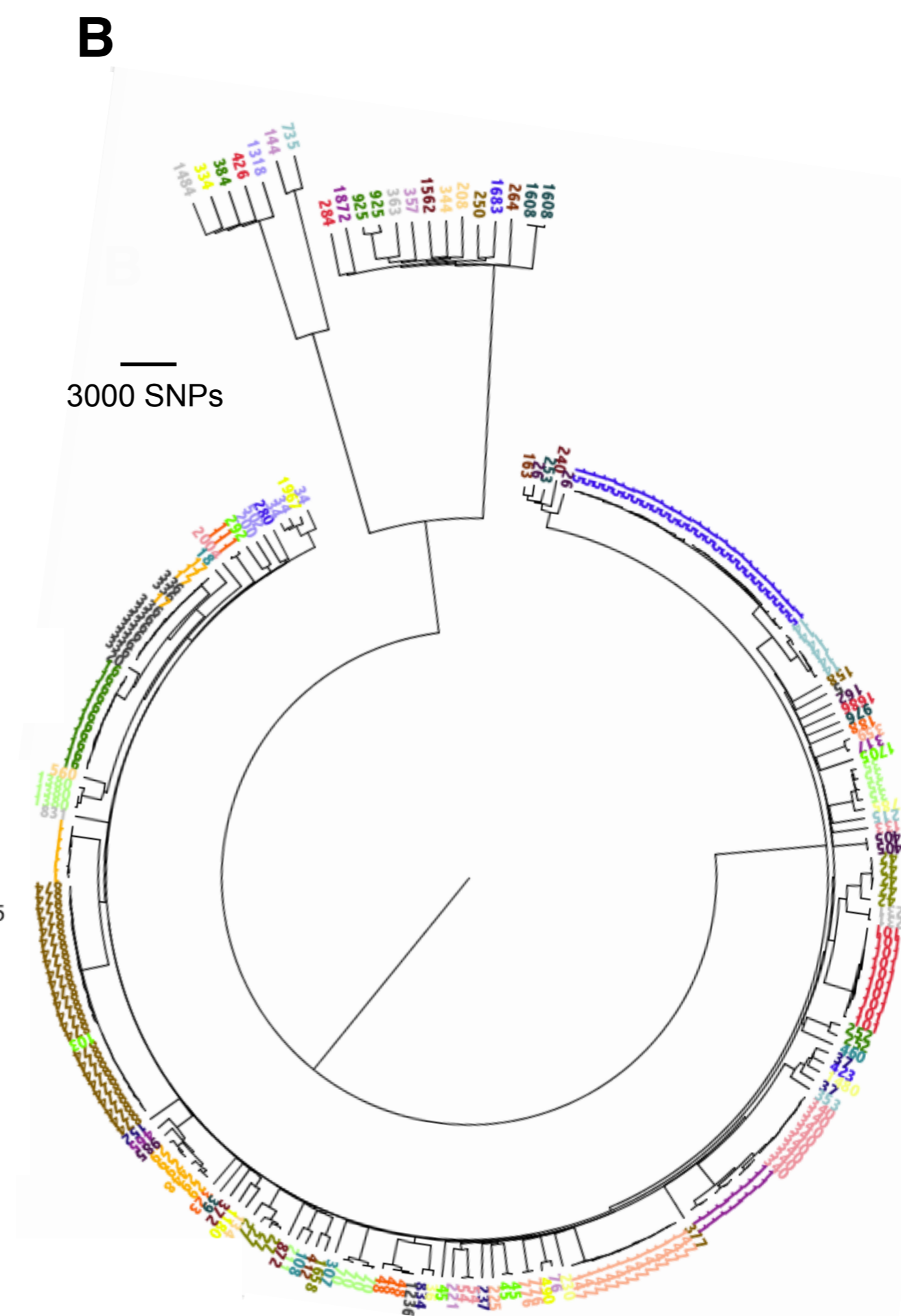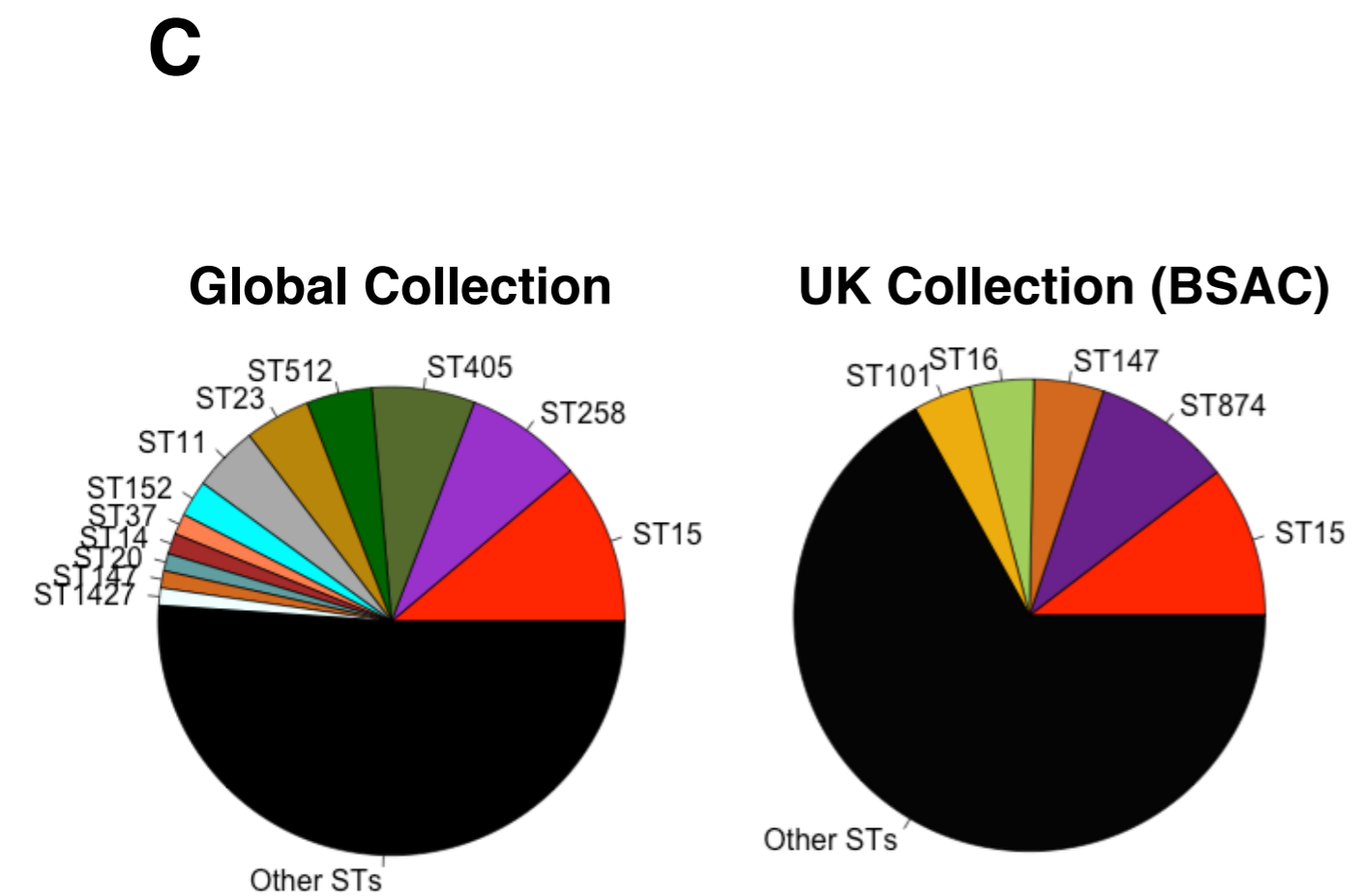

Supplement: FIG S1 [file mbo001173200sf1.pdf]

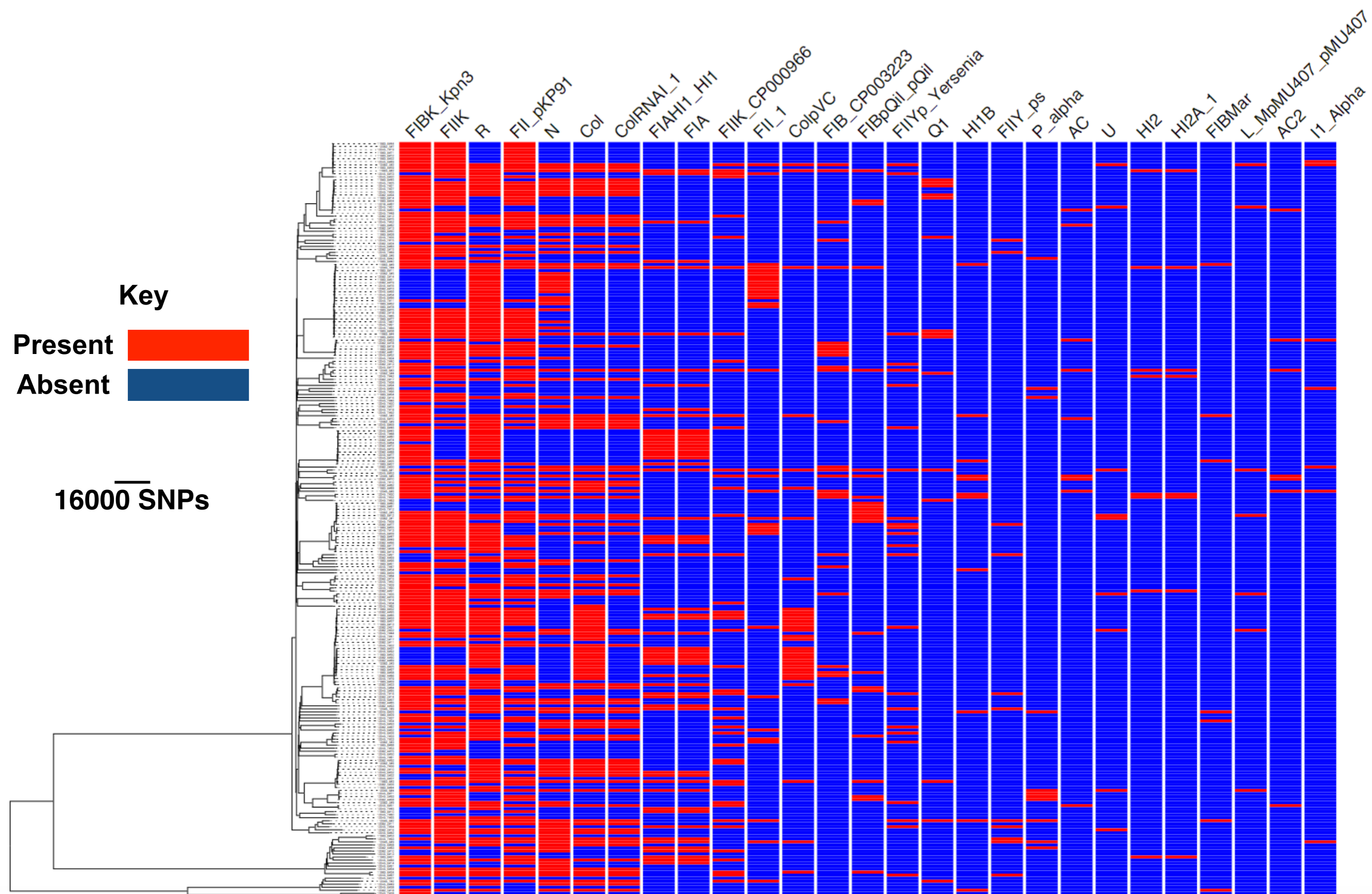

Supplement: FIG S2 [file mbo001173200sf2.pdf]

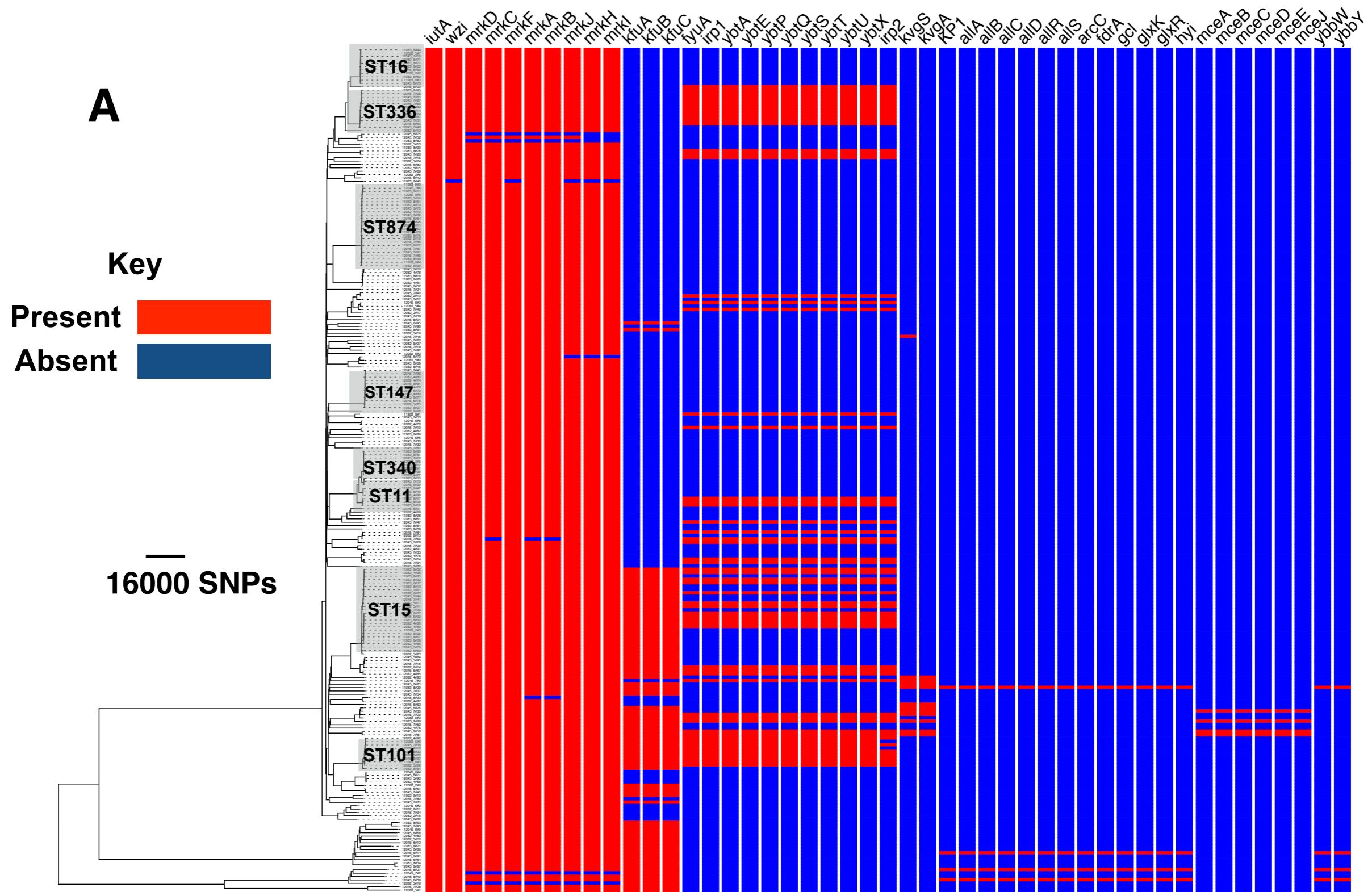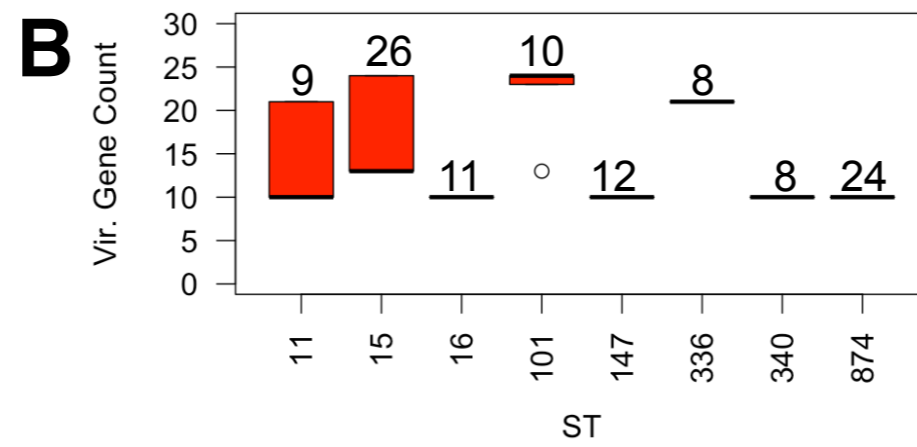

Supplement: FIG S3 [file mbo001173200sf3.pdf]

cip

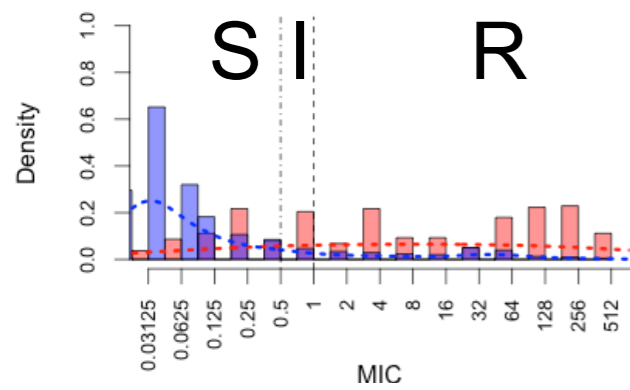

Key

EUCAST

BSAC

amc

ctx

gen

tet

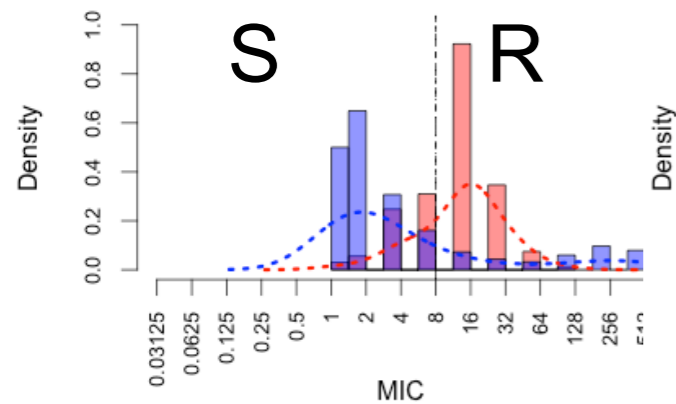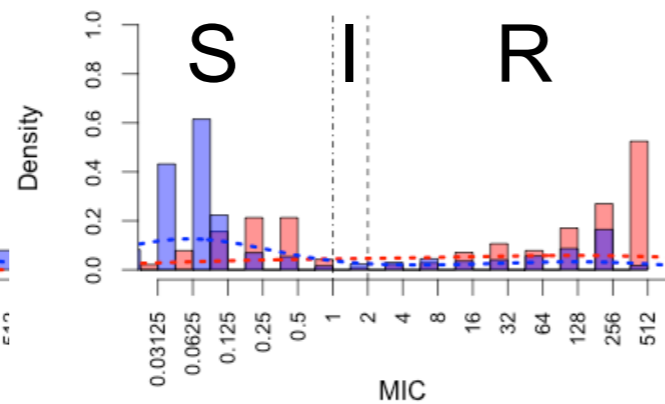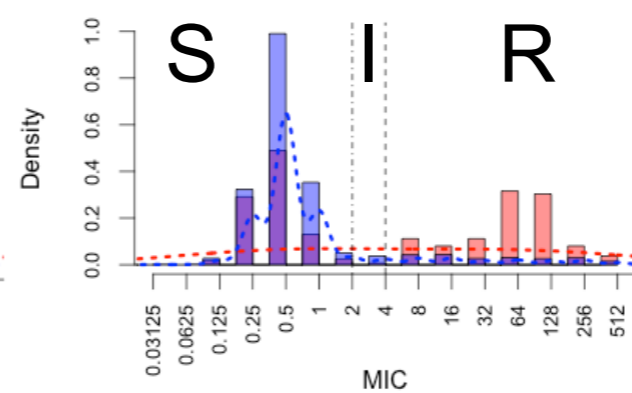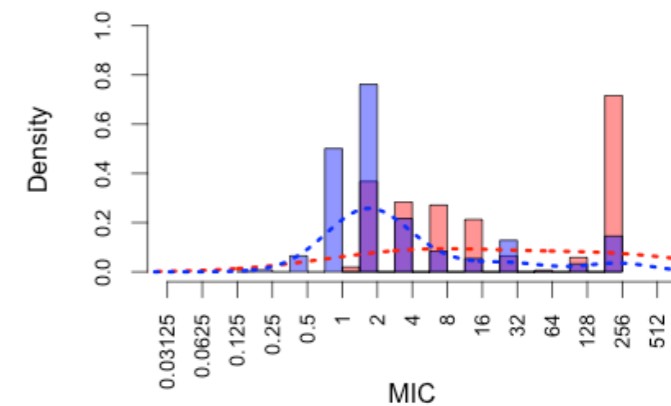

caz

cxm

ipm

tgc

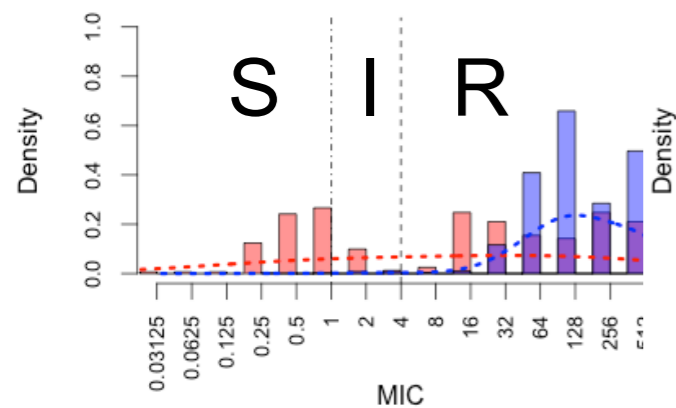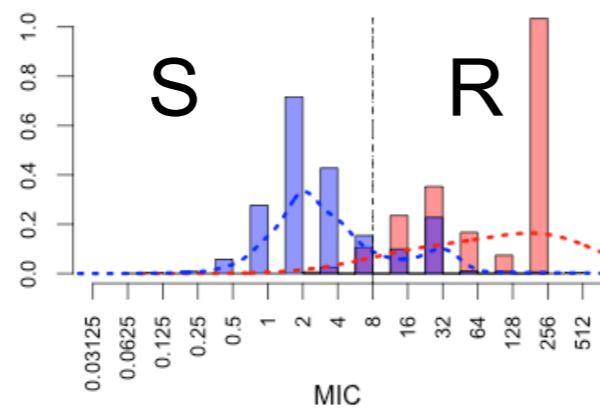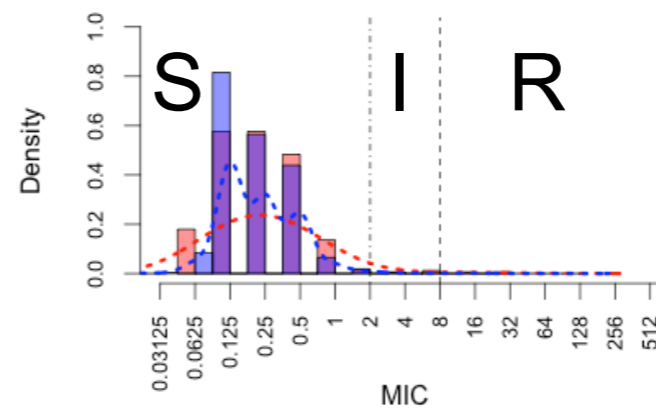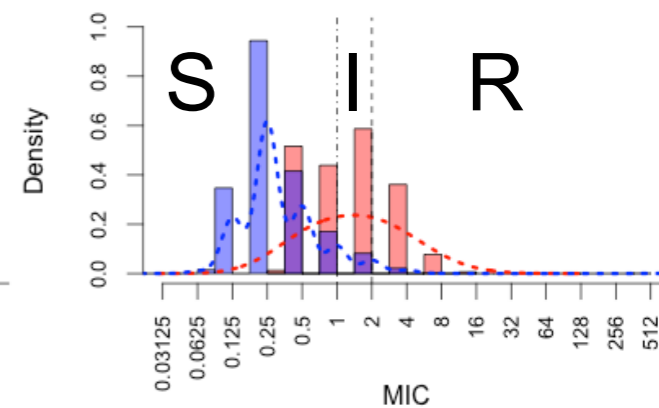

amx

fox

min

tzp

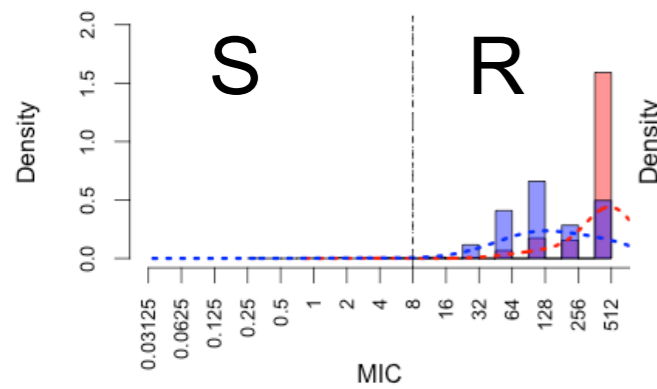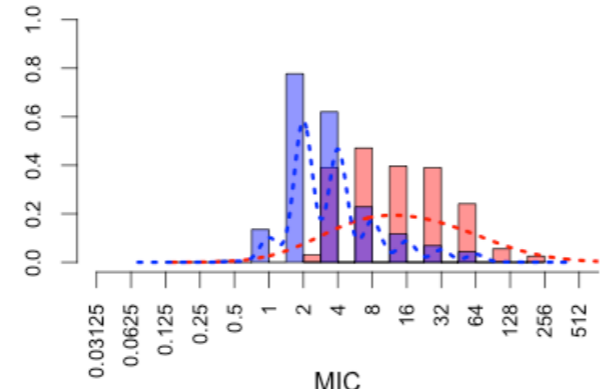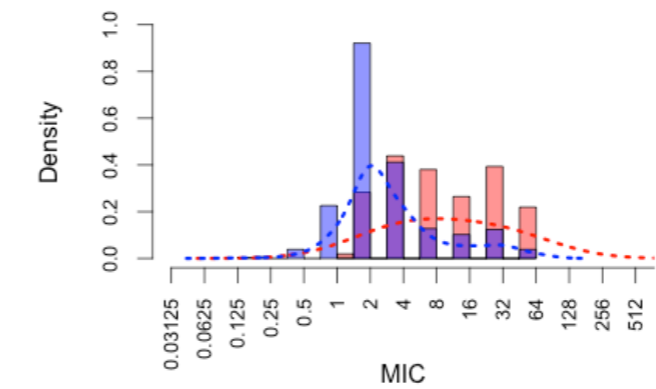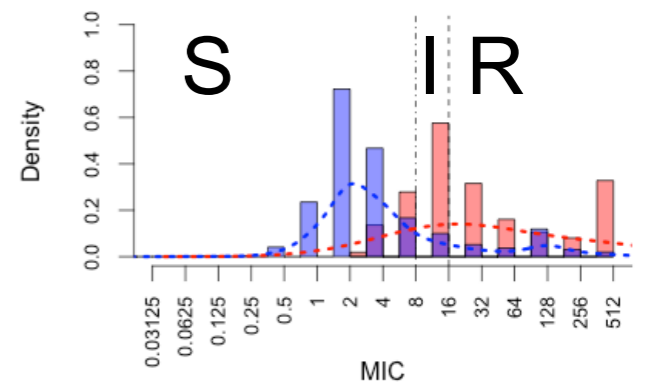

Supplement: FIG S4 [file mbo001173200sf4.pdf]

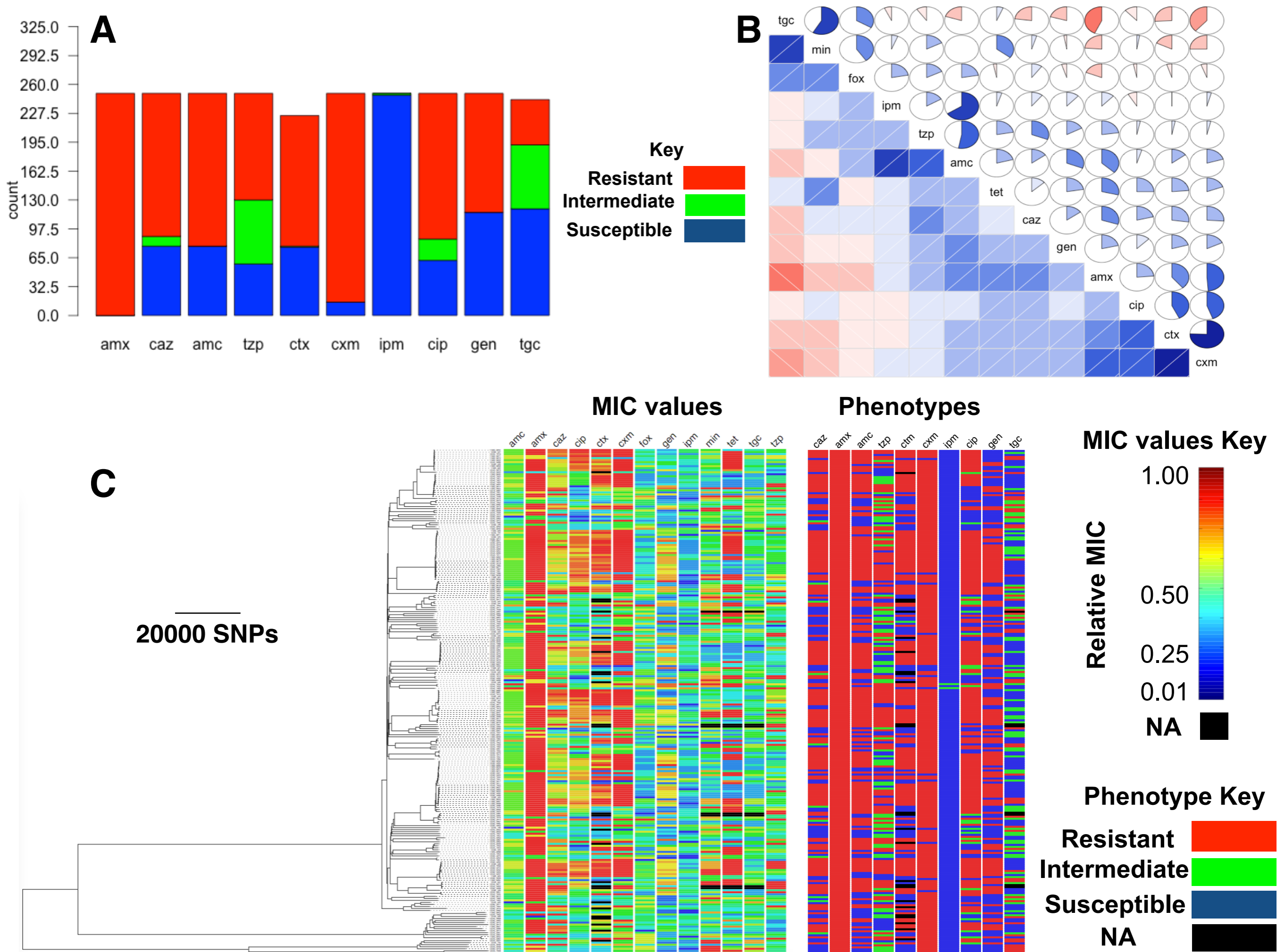

**D****amx**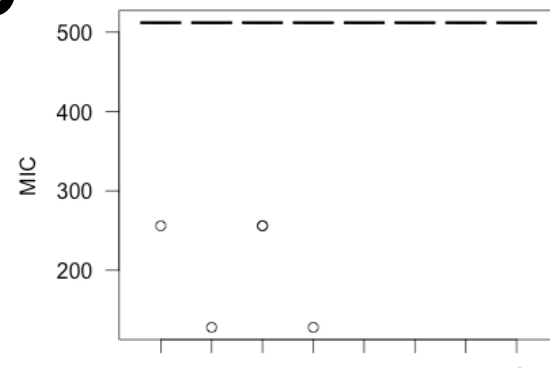**amc**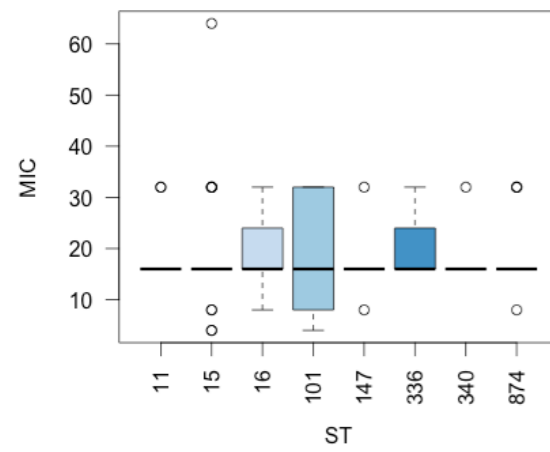**caz**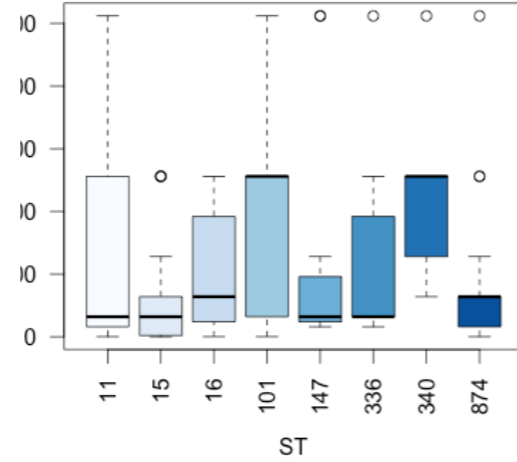**fox**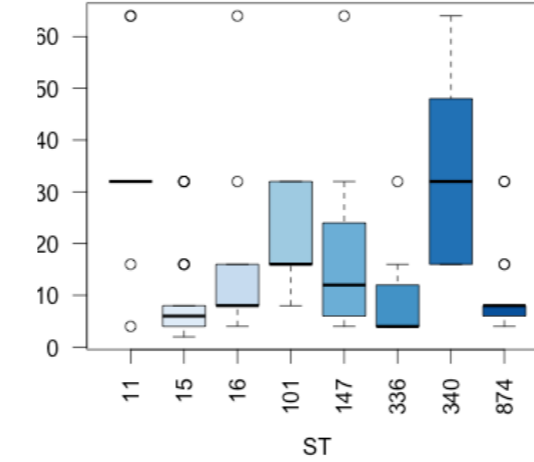**min**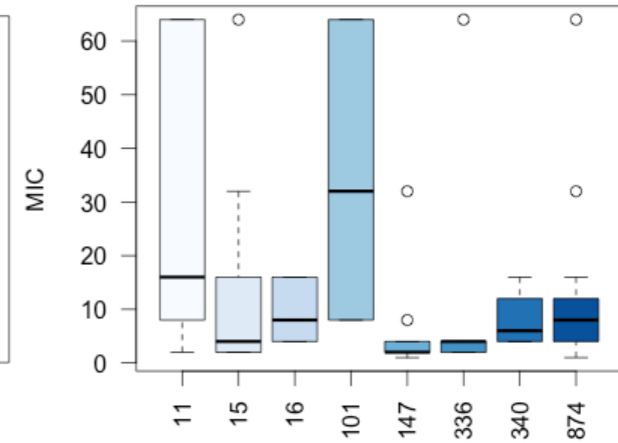**cip**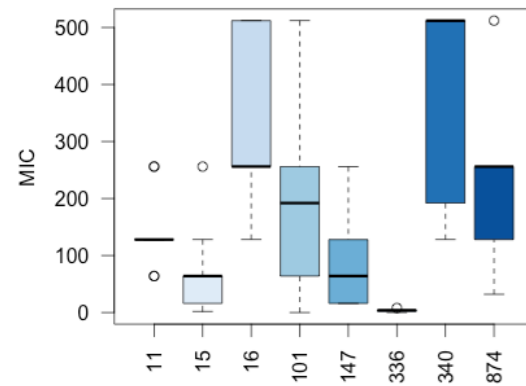**ctx**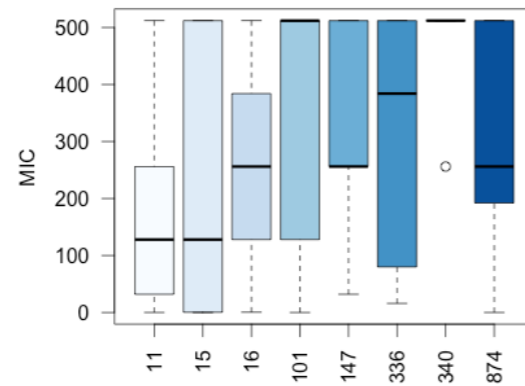**gen**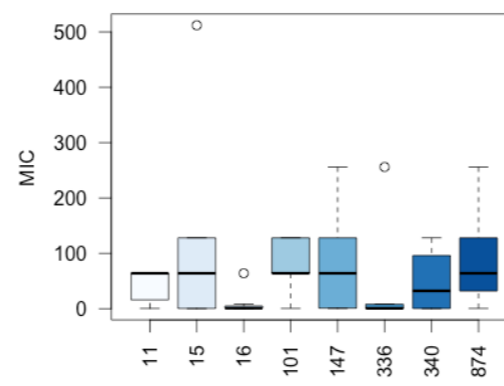**tet**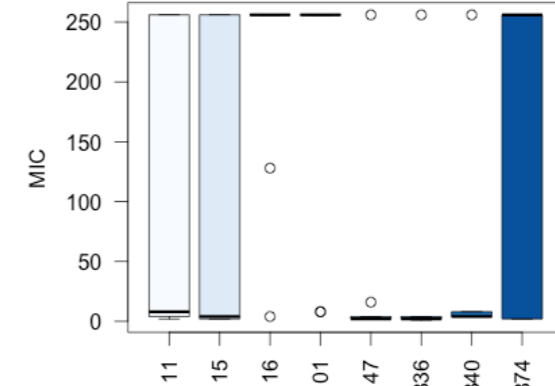**tzp**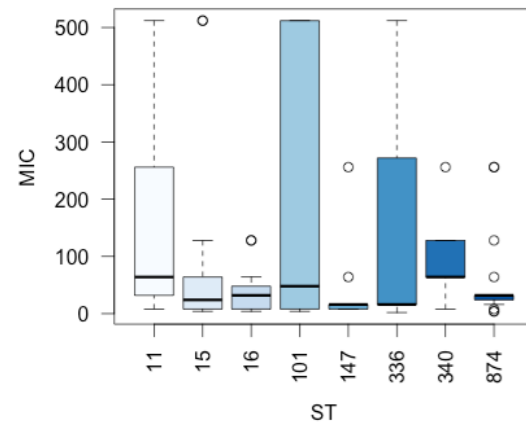**cxm**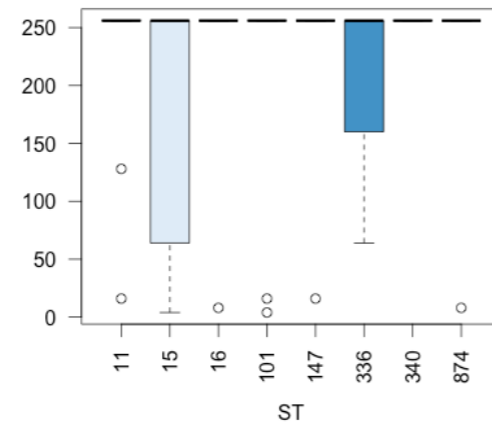**ipm**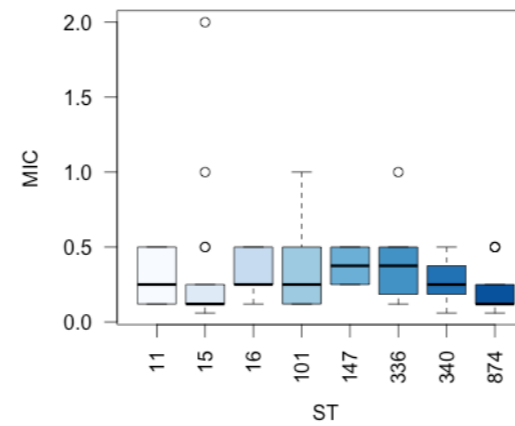**tgc**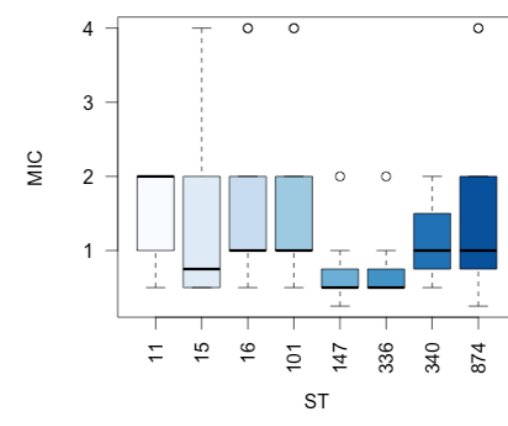

E

ipm

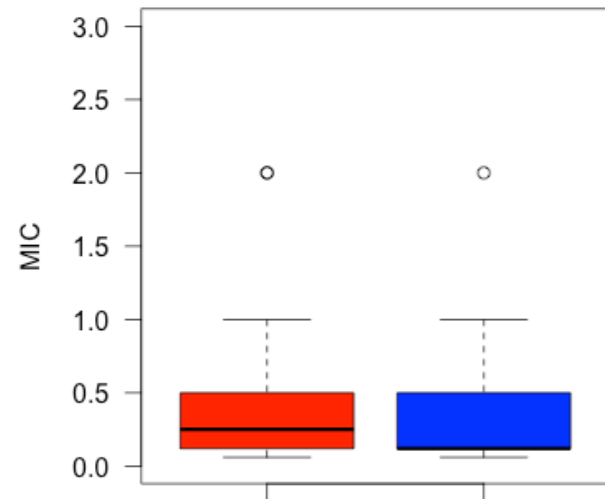

amc

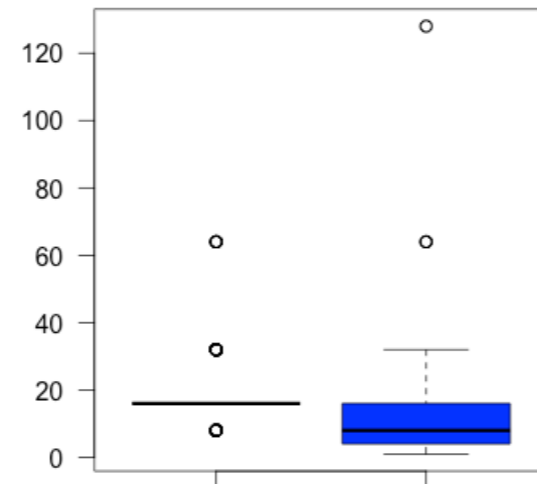

amx

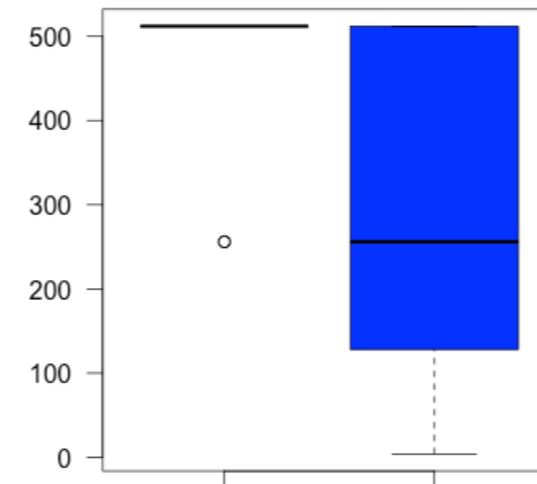

caz

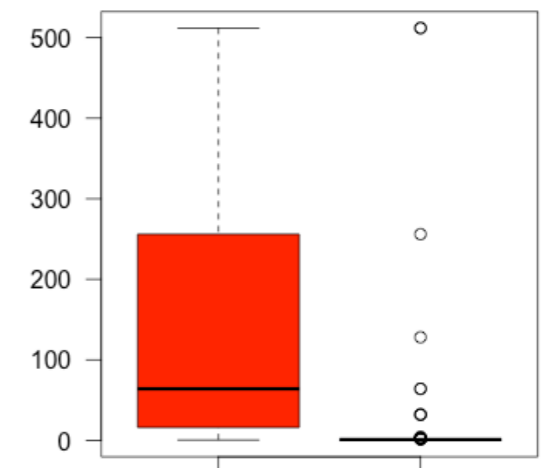

tzp

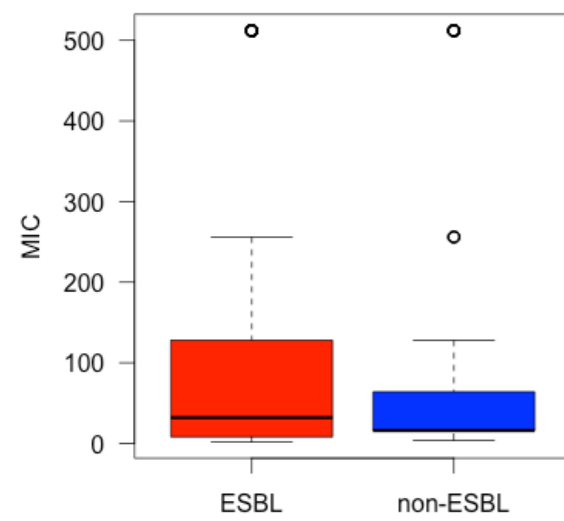

cxm

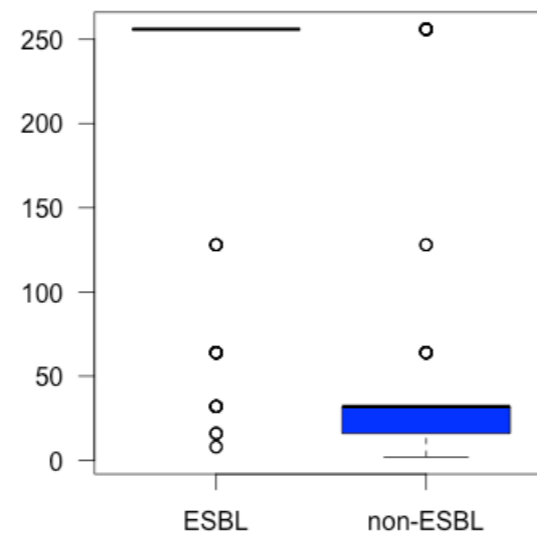

ctx

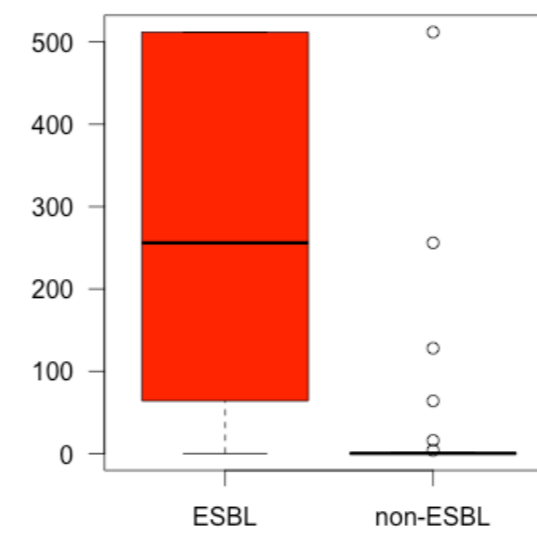

fox

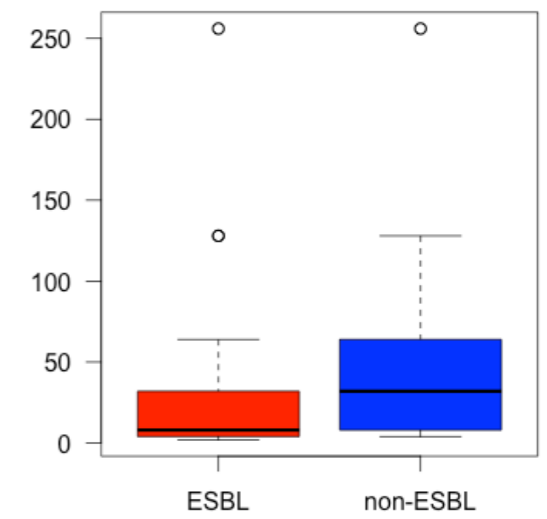

Supplement: FIG S5 [file mbo001173200sf5.pdf]

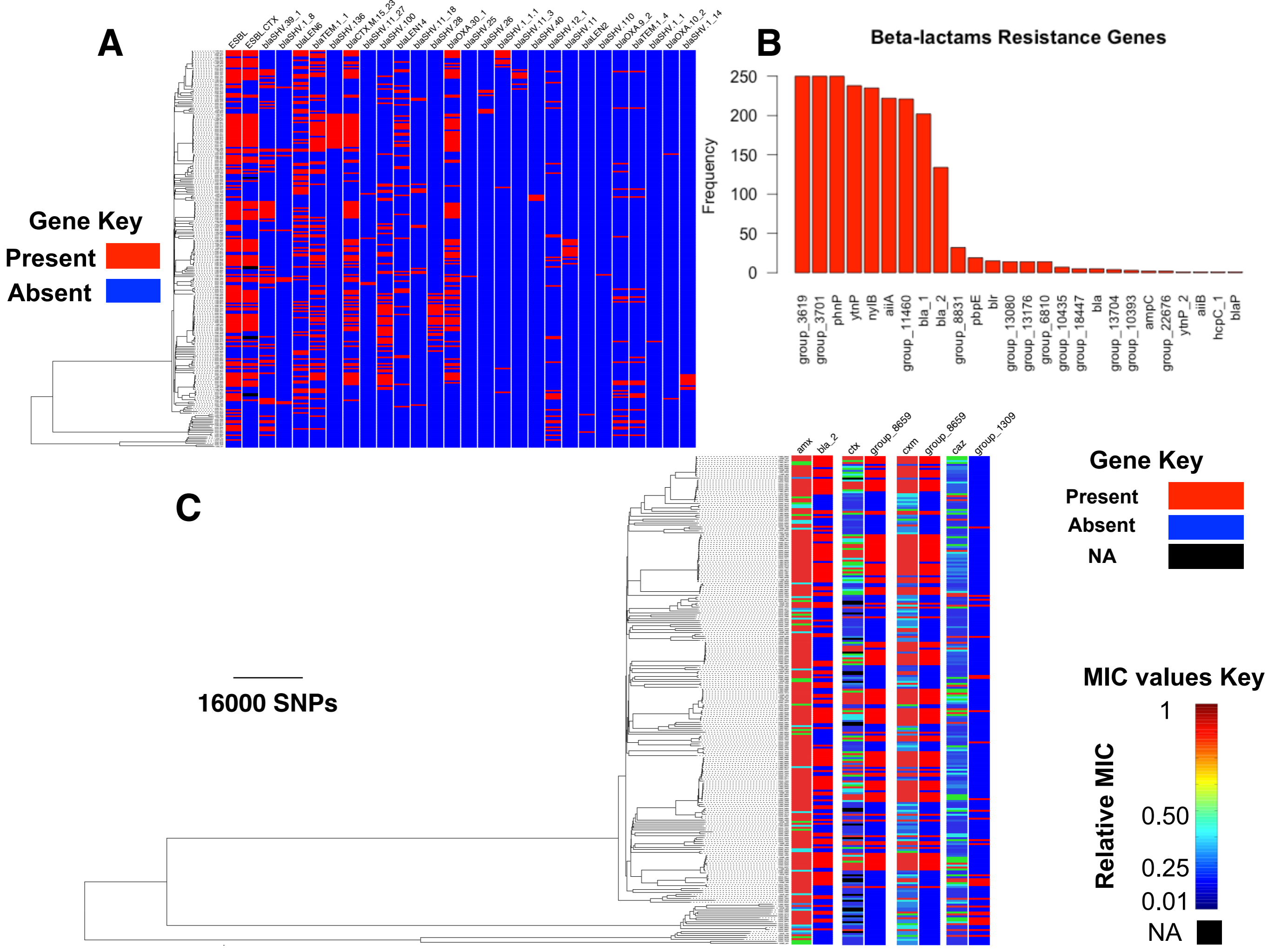

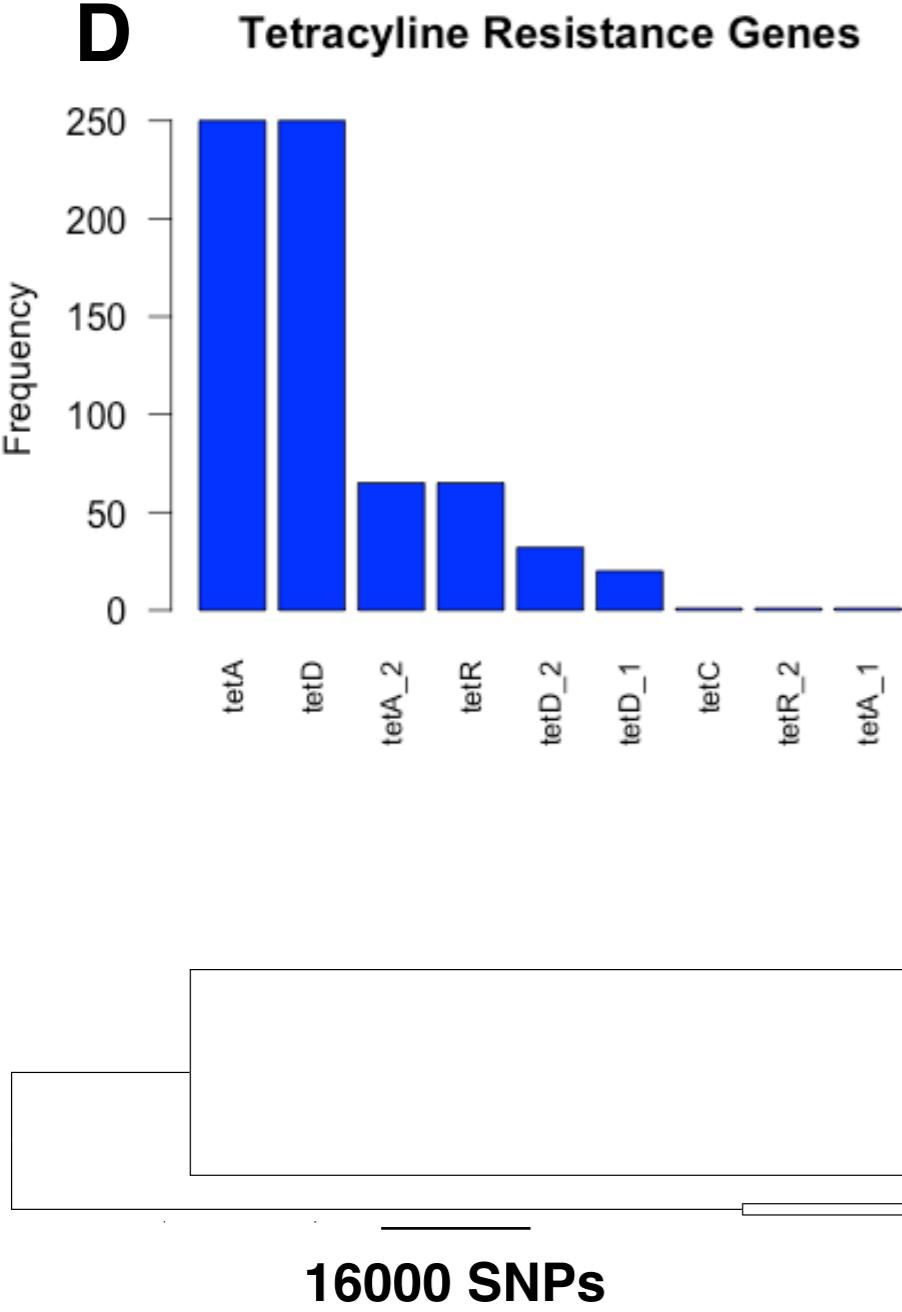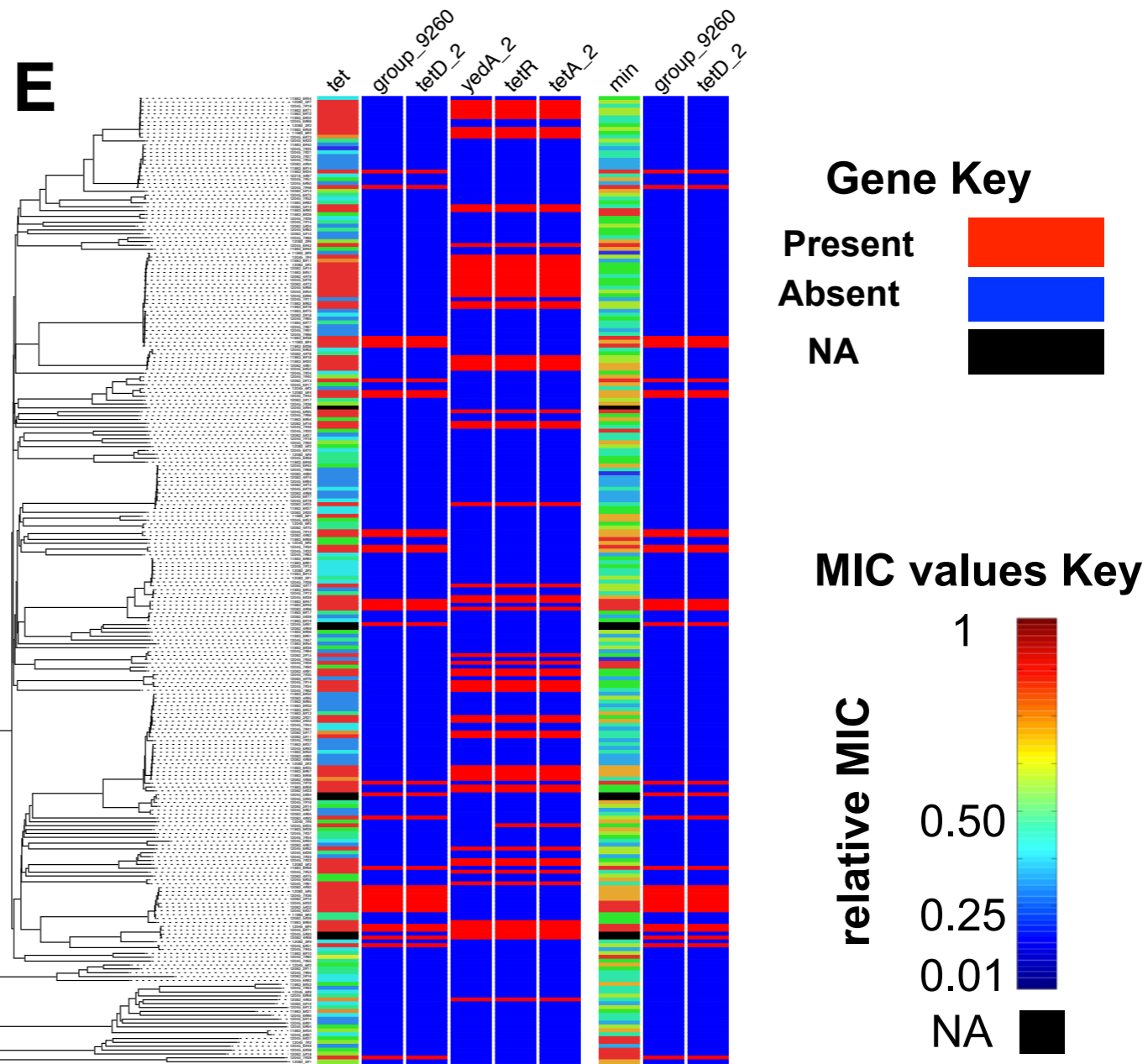

Supplement: FIG S6 [file mbo001173200sf6.pdf]

**A**

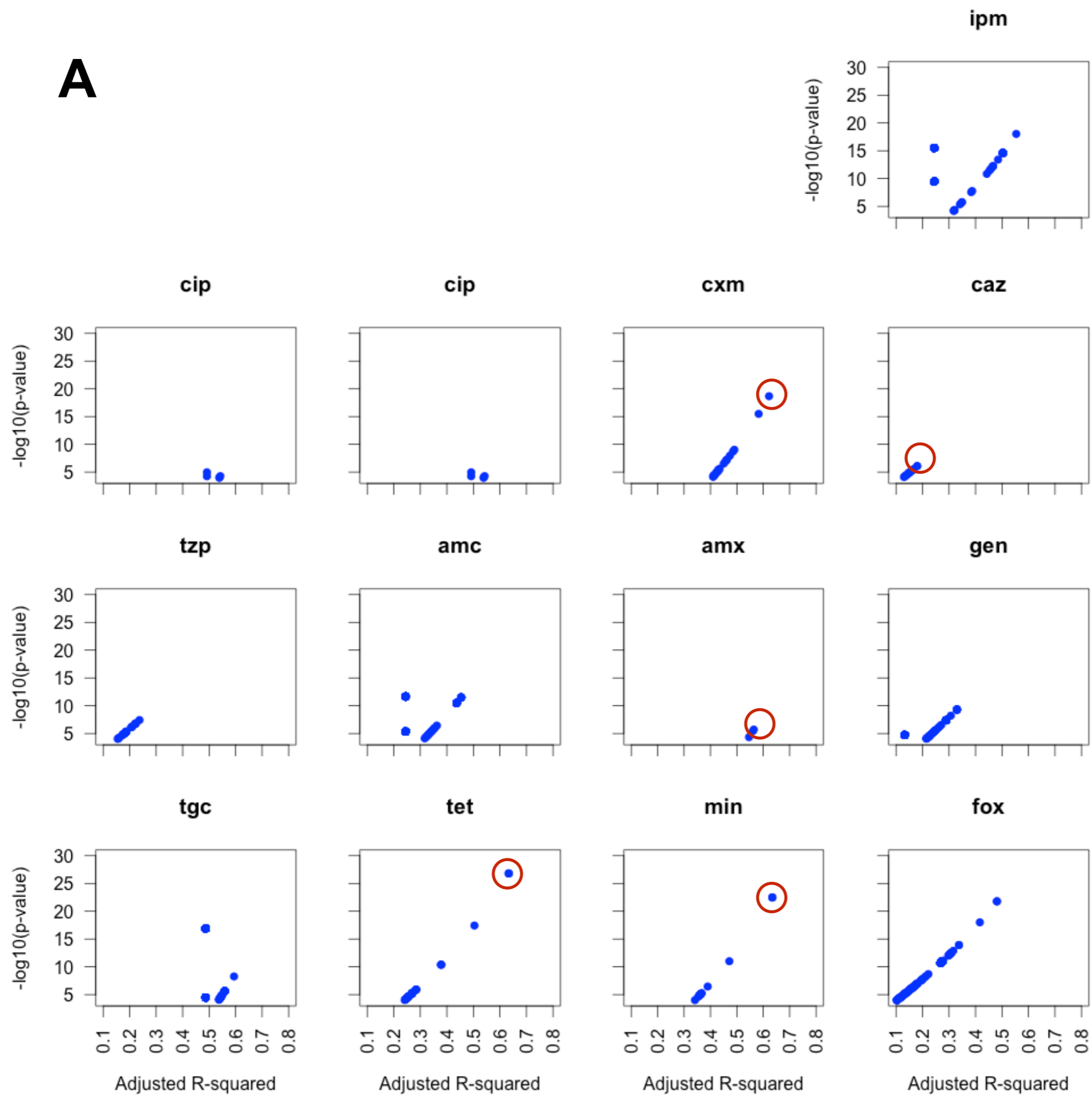

**B**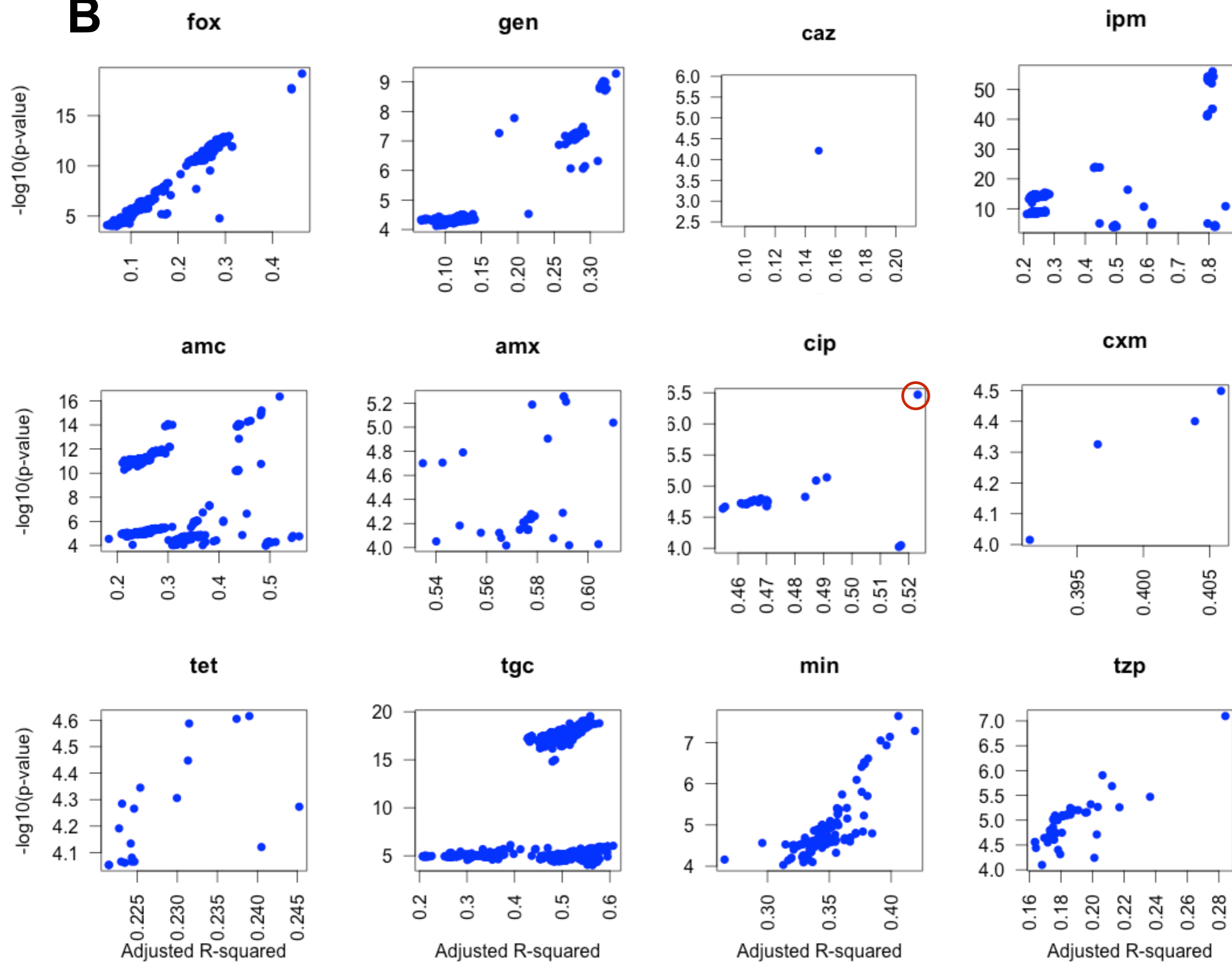

Supplement: FIG S7 [file mbo001173200sf7.pdf]
